# Targeting mTOR and survivin concurrently potentiates radiation therapy in renal cell carcinoma by suppressing DNA damage repair and amplifying mitotic catastrophe

**DOI:** 10.1186/s13046-024-03079-8

**Published:** 2024-06-06

**Authors:** Hari K. Rachamala, Vijay S. Madamsetty, Ramcharan S. Angom, Naga M. Nakka, Shamit Kumar Dutta, Enfeng Wang, Debabrata Mukhopadhyay, Krishnendu Pal

**Affiliations:** 1 Department of Biochemistry and Molecular Biology, Mayo Clinic Florida, 4500 San Pablo Road S, Jacksonville, FL 32224 USA; 2 Present Address: PolyARNA Therapeutics, One Kendal Square, Cambridge, MA 01329 USA

**Keywords:** Renal cancer, Radiation therapy, mTOR, Survivin, Mitotic catastrophe

## Abstract

**Background:**

Renal cell carcinoma (RCC) was historically considered to be less responsive to radiation therapy (RT) compared to other cancer indications. However, advancements in precision high-dose radiation delivery through single-fraction and multi-fraction stereotactic ablative radiotherapy (SABR) have led to better outcomes and reduced treatment-related toxicities, sparking renewed interest in using RT to treat RCC. Moreover, numerous studies have revealed that certain therapeutic agents including chemotherapies can increase the sensitivity of tumors to RT, leading to a growing interest in combining these treatments. Here, we developed a rational combination of two radiosensitizers in a tumor-targeted liposomal formulation for augmenting RT in RCC. The objective of this study is to assess the efficacy of a tumor-targeted liposomal formulation combining the mTOR inhibitor everolimus (E) with the survivin inhibitor YM155 (Y) in enhancing the sensitivity of RCC tumors to radiation.

**Experimental design:**

We slightly modified our previously published tumor-targeted liposomal formulation to develop a rational combination of E and Y in a single liposomal formulation (EY-L) and assessed its efficacy in RCC cell lines in vitro and in RCC tumors in vivo. We further investigated how well EY-L sensitizes RCC cell lines and tumors toward radiation and explored the underlying mechanism of radiosensitization.

**Results:**

EY-L outperformed the corresponding single drug-loaded formulations E-L and Y-L in terms of containing primary tumor growth and improving survival in an immunocompetent syngeneic mouse model of RCC. EY-L also exhibited significantly higher sensitization of RCC cells towards radiation in vitro than E-L and Y-L. Additionally, EY-L sensitized RCC tumors towards radiation therapy in xenograft and murine RCC models. EY-L mediated induction of mitotic catastrophe via downregulation of multiple cell cycle checkpoints and DNA damage repair pathways could be responsible for the augmentation of radiation therapy.

**Conclusion:**

Taken together, our study demonstrated the efficacy of a strategic combination therapy in sensitizing RCC to radiation therapy via inhibition of DNA damage repair and a substantial increase in mitotic catastrophe. This combination therapy may find its use in the augmentation of radiation therapy during the treatment of RCC patients.

**Supplementary Information:**

The online version contains supplementary material available at 10.1186/s13046-024-03079-8.

## Background

Kidney cancer is one of the ten most prevalent cancers in the United States, ranking as the sixth and ninth most common cancer in men and women, respectively [1]. In 2023, it is anticipated that around 81,800 new cases of kidney cancer will be diagnosed in the United States, resulting in 14,890 deaths [1]. Renal cell carcinoma (RCC) accounts for approximately 90% of all kidney cancer cases [2]. While early-stage RCC patients have a better prognosis, the survival rate for advanced-stage RCC patients is dismal, with a five-year survival rate of 12-15% only [1]. One-third of RCC patients present with widespread metastasis at diagnosis, and nearly half of the patients who undergo primary tumor resection develop distant metastasis [3]. Existing therapies for advanced RCC, including chemotherapy, radiotherapy, and targeted therapies such as tyrosine kinase inhibitors (TKI), mammalian target of rapamycin (mTOR) inhibitors, or vascular endothelial growth factors (VEGF)-targeted therapies are unable to provide long-term survival benefits [4]. Recently, immune checkpoint inhibitors (ICI) have been approved for the treatment of advanced RCC, either alone or in combination with TKI, following promising results in large Phase III trials [5–8]. Nonetheless, alternative therapies are necessary for patients who suffer from severe side effects, experience disease progression after an initial positive response, or fail to respond altogether to ICI [9].

Apart from immunotherapy, radiation therapy (RT) is another effective curative treatment method for cancer [10]. However, different types of cancer have varying degrees of resistance to RT, with RCC being known to have relatively higher resistance compared to other cancer types [11, 12]. Cancer cells develop resistance to RT through various mechanisms, including DNA damage repair, cell cycle arrest, changes in oncogenic and tumor suppressor signaling pathways, tumor microenvironment (TME) remodeling, cancer stemness, and metabolic reprogramming [13]. However, recent advancements in treatment planning, delivery techniques, immobilization strategies, image guidance, and computed tomography have substantially enhanced the effectiveness of RT. Assisted by modern computing power, single-fraction and multi-fraction stereotactic ablative radiotherapy (SABR) have achieved greater precision in delivering high-dose radiation, resulting in better treatment outcomes while minimizing treatment-related toxicities [14]. Consequently, numerous clinical trials are currently investigating the effectiveness of SABR, either alone or in combination with other treatment modalities, as viable treatment options for RCC [12]. However, combining SABR with agents that can override RCC’s intrinsic resistance to RT is more likely to improve therapeutic outcomes. Several studies have already demonstrated that certain therapeutic agents, including chemotherapy, can act as radiosensitizers, thereby prompting research studies combining RT with such agents [15].

In this study, we have proposed to use the combination of everolimus, an mTOR inhibitor, with YM155, a survivin inhibitor, to sensitize RCC tumors toward radiation. Our interest in including everolimus as a radiosensitizer in our study stems from its inherent antitumor, antiangiogenic, and radiosensitizing effect in different cancer types including RCC [16–23], and the fact that it was approved by the FDA for the treatment of advanced RCC as a second-line therapy. Notably, mTOR inhibitors disrupt multiple mechanisms associated with radioresistance in cancer cells, including cancer stemness, metabolic pathways, DNA damage repair pathways, and various oncogenic pathways [18]. Consequently, several clinical trials investigated the efficacy of combining RT with everolimus across various cancer types, including RCC [19–23]. While this approach demonstrated efficacy in some patients, its overall clinical significance was compromised by dose-limiting toxicities [19, 24, 25].

Nonetheless, the eventual emergence of resistance to mammalian target of rapamycin (mTOR) inhibitors among RCC patients led us to explore combinations with other agents capable of overcoming such resistance. Given that survivin inhibitor YM155 demonstrated efficacy in surmounting resistance to mTOR inhibitors in renal cancer [26], it emerged as a logical selection for combination in our investigation. Furthermore, YM155 exhibits notable antitumor, antiangiogenic, and radiosensitizing activities across various cancer types as well [27, 28]. Survivin expression has been found to be associated with RT resistance, and genetic depletion or chemical inhibition of survivin has been shown to enhance radiosensitivity across various cancer types [27, 29–32]. Survivin is implicated in multiple RT resistance mechanisms including DNA damage repair, cell cycle, metabolic reprogramming, and stemness [33–35]. Interestingly, despite being tested in numerous clinical trials, YM155 has not yet received approval for clinical use [36]. The lack of success in clinical trials may be attributed to its poor pharmacokinetic stability, as indicated by studies revealing a rapid decline in YM155 levels in both serum and tumors after completing treatment [37].

Nonetheless, based on these observations, we postulated that YM155 would synergize with everolimus in sensitizing RCC cells to RT. To our knowledge, no prior investigation has examined the potential synergistic effects of combining these two agents toward radiosensitization. This knowledge gap prompted us to test their combined efficacy in sensitizing RCC toward radiation. However, to mitigate any potential increase in toxicity due to the combination therapy, which often compels treatment discontinuation or dose reductions [38], we employed a target-specific drug delivery platform capable of delivering multiple drugs simultaneously to tumors, a strategy being explored for different drug combinations [39]. Previously we developed a tumor-targeted liposomal formulation that showed promise in delivering multiple drugs to tumors effectively without eliciting toxicity in animal models [40, 41]. We hypothesized that a similar tumor-targeted liposomal formulation combining everolimus with YM155 will have better efficacy and reduced systemic toxicity and will synergistically sensitize RCC tumors towards RT. The goal of this study is to determine whether this tumor-targeted liposomal formulation combining everolimus and YM155 inhibits growth in RCC tumors and at the same time sensitizes them to radiation therapy.

## Methods

### Reagents

DOPC and DSPE-PEG(2000)-OMe were purchased from Avanti Polar Lipids and Nanosoft Polymers, respectively. Cholesterol was purchased from Sigma. TTP-conjugated lipopeptide was synthesized as described previously [40]. Everolimus and cabozantinib were obtained from LC laboratories. YM155 and sunitinib were obtained from MedChemExpress and Selleckchem, respectively. Antibodies against mTOR, phospho-mTOR, p70S6K, phospho-p70S6K, survivin, ATM, PARP1, CD4, and β-actin were obtained from Cell Signaling Technology. ATR, Chk1, and Chk2 antibodies were obtained from Santa Cruz Biotechnology. CD3, CD8, and Ki67 antibodies were from Abcam, while CD45 antibody was from Biolegend. The phospho-p70S6K antibody used for immunohistochemistry was from Invitrogen. Purified anti-PD-1 antibody [42] was a kind gift from Dr. Keith L. Knutson (Mayo Clinic) who obtained it from the Antibody Core Facility at Mayo Clinic (Rochester, MN).

### Cell culture

786-O cell line was obtained from the American Type Culture Collection (ATCC). Luciferase-labeled Renca cell lines was a kind gift from Thomas S. Griffith [43]. The authors did not authenticate the cell lines. 786-O cell line was maintained in Dulbecco’s Modified Eagle Medium (DMEM) and RPMI-1640 medium was used for maintaining Renca cell lines. Both the media were supplemented with 10% FBS and 1% penicillin–streptomycin (Invitrogen) and cells were cultured at 37 °C in a humidified atmosphere with 5% CO_2_. Cells from 85 to 90% confluent cultures were used in the experiments.

### Preparation and characterization of drug-loaded liposomes

A modified ethanol injection technique was employed to formulate the E-L, Y-L, or EY-L liposomes [44]. Briefly, required amounts of DOPC (3.93 mg), Cholesterol (0.483 mg), DSPE-PEG(2000)-OMe (0.27 mg), and TTP-conjugated lipopeptide (0.22 mg) with everolimus (0.4 mg), and/or YM155 (0.8 mg) were dissolved in 400 µL ethanol and the solution was warmed at 65 °C for 5 min. Subsequently, this ethanolic solution was slowly injected into 600 µL preheated milli-Q water at 65 °C while continuously vortexing the mixture, resulting in the spontaneous formation of liposomes. Removal of unentrapped drugs and liposome characterization were performed as described previously [40, 41].

### In vitro cytotoxicity assay

Approximately 5 × 10^3^ 786-O or Renca cells per well were seeded in 96-well plates and allowed to settle for 18–24 h. Then, cells were treated with increasing concentrations of E-L, Y-L, and EY-L diluted in respective media and incubated for 72 h (*n* = 4 wells per concentration). Cell viability was determined with Celltiter 96 Aqueous One Solution Cell Proliferation Assay kit (Promega) as described previously [40, 41]. IC50 values were estimated by non-linear curve fitting using GraphPad Prism (v 9.4.0). Similar experiments were performed using sunitinib and cabozantinib.

### In vitro radiosensitivity experiments

For in vitro radiosensitivity, RCC cells were plated in 2 sets of 6 well plates and treated with PBS, E-L, Y-L, and EY-L for 48 h. The sub-IC50 concentration of liposomes (0.01% for 786-O, 0.125% for Renca) was selected based on the results from the MTS assay to minimize cell death due to drug treatment only. One set of cells was then exposed to 2 Gy radiation at room temperature at a 3.9 Gy/min dose rate and a 160 kV tube voltage using an X-RAD 160 Irradiator (Precision X-Ray Inc., USA). Following irradiation, the cell samples were returned to a 5% CO_2_ incubator. Both irradiated and non-irradiated cells were then harvested and seeded in triplicates (100 cells/well) in 12-well plates in fresh culture media without drugs and allowed to grow for 10–14 days. Then, colonies were fixed with 4% formaldehyde and stained with 0.2% Crystal Violet solution, and colonies larger than 50 cells were counted. The surviving fraction for a particular treatment group was determined by dividing the plating efficiency of the irradiated cells by the plating efficiency of the corresponding unirradiated cells. Bliss independent principle was considered to determine the synergistic effect of EY-L over E-L and Y-L in inducing radiosensitivity in RCC cells [45, 46]. Briefly, if E_a_ and E_b_ are the observed effects with drug A alone at dose a, and drug B alone at dose b, the Bliss predicted effect of the combination dose (a, b) of drugs A and B respectively can be calculated from the formula: E_a_ + E_b_ − E_a_E_b_, where 0 ≤ E_a_ ≤ 1 and 0 ≤ E_b_ ≤ 1.The combination index (CI), calculated using the ratio (E_a_ + E_b_ − E_a_E_b_) / E_ab_, where E_ab_ is the observed effect at the combination dose (a, b) of drug A and drug B, indicates synergy, antagonism, or additivity when CI is under, above, or equal to 1, respectively.

### Immunoblot analysis

Lysates were prepared from treated cells using NP-40 lysis buffer (Boston BioProducts) supplemented with a protease inhibitor cocktail (Sigma) and Halt phosphatase inhibitor (ThermoFisher Scientific). Protein concentrations of the lysates were measured by either Bradford assay (Bio-Rad) or Pierce BCA Protein Assay kit (ThermoFisher Scientific). Equal amounts of proteins from each sample were subjected to SDS-PAGE and transferred to polyvinyl difluoride membranes followed by immunoblotting with primary antibodies (1:1000 dilutions for mTOR, phospho-mTOR, p70S6K, phospho-p70S6K, survivin, ATM, and PARP1 and 1:250 dilutions for ATR, Chk1, and Chk2) and respective secondary antibodies (1:10000). Enzyme-linked chemiluminescence was used to detect antibody-reactive bands in Chemidoc MP (Bio-Rad). Blots from the same experiments were used for presentation.

### Animals used in the study

Six- to eight-week-old SCID and Balb/c mice were obtained from in-house breeding and housed in the institutional animal facilities. All animal experiments were performed following the Association for Assessment and Accreditation of Laboratory Animal Care (AAALAC) guidelines under protocols approved by the Mayo Clinic Institutional Animal Care and Use Committee (IACUC).

### In vivo tumor regression experiment in subcutaneous renca tumors

The in vivo tumor regression efficacy of the drug-loaded liposomes was analyzed in syngeneic subcutaneous Renca tumors developed in Balb/c mice (*n* = 5 per treatment group). For subcutaneous tumor cell implantation, Balb/c mice were anesthetized with intraperitoneal (i.p.) administration of ketamine/xylazine. The fur on the right flank of the mice was shaved and the shaved area was disinfected with iodine solution and 70% alcohol. Then 1 × 10^6^ Renca cells suspended in 100 µl sterile PBS was injected subcutaneously into the shaved area of each mouse using a 1 ml syringe equipped with a 26-gauge needle. Following injection, mice were transferred to their cage and kept under a heating lamp for recovery. Mice were observed daily, and treatment was started when the average tumor volume reached ~ 100 mm^3^. E-L (1.94 mg/kg E), Y-L (1.44 mg/kg Y), and EY-L (1.94 mg/kg E, 1.44 mg/kg Y) were intravenously administered twice a week for 4 weeks. Tumors were measured weekly with calipers and tumor volumes were calculated using the formula: Volume = 0.5 x a x b^2^ where a and b are the longest and shortest diameter, respectively. Tumor growth curves were obtained by plotting tumor volumes against time. Finally, mice were sacrificed to harvest the tumors for immunohistochemistry. The efficacy of a mouse anti-PD-1 antibody (αPD-1, 200 µg/mouse, every three to four days for a total of six treatments) was evaluated in a similar experiment for comparison.

### In vivo tumor regression experiment in orthotopic renca tumors

We further analyzed the efficacy of EY-L in syngeneic orthotopic Renca tumors developed in Balb/c mice (*n* = 4 for control and *n* = 5 for EY-L treatment group). For orthotopic tumor cell implantation, Balb/c mice were anesthetized with i.p. administration of ketamine/xylazine before any surgical procedures. The fur on the left dorsal area was shaved, and the shaved area was sterilized with iodine solution and 70% alcohol. A 1 cm incision was made into the body wall parallel to the spine and then slightly widened with forceps to allow for the kidney to be exteriorized. Approximately 1 × 10^5^ luciferase-transfected Renca cells in 50 µl sterile PBS were injected into the kidney capsule. The kidney was replaced into the body cavity and the body wall and skin were sutured. Following injection, mice were transferred to their cage and kept under a heating lamp for recovery. Analgesia was given to the mice from 2 days pre-surgery to 3 days post-surgery. Mice were observed daily, and treatment started 2 weeks after implantation. EY-L (1.94 mg/kg E, 1.44 mg/kg Y) was intravenously administered twice a week for 4 weeks. Tumor growth was monitored weekly by measuring bioluminescence in an IVIS Xenogen (Perkin Elmer). For bioluminescence imaging, orthotopic tumor-bearing mice were intraperitoneally injected with 150 mg/kg D-Luciferin and kept in an anesthesia chamber connected to isoflurane flow. Once the mice became unconscious, they were kept side by side inside IVIS Xenogen chamber under continuous anesthesia using nosecones and imaged for bioluminescence signal with 1 s exposure. Tumor growth curves were obtained by plotting fold changes in bioluminescence from initial values against time. The survival was also analyzed by monitoring the IACUC-approved endpoint for each mouse.

### In vivo radiosensitivity experiments

To evaluate the in vivo radiosensitization potential of EY-L in RCC tumors, we first developed subcutaneous 786-O xenografts by implanting 2 × 10^6^ cells into the right flanks of 6–8 weeks old SCID mice. After 30 days, when the average tumor volume reached 25 mm^3^, twice-a-week EY-L (1.94 mg/kg E, 1.44 mg/kg Y) intravenous administrations were started and continued for 3 weeks. Two doses of focused single-beam 10 Gy radiation each were administered to the tumors on days 12 and 19 for mice belonging to the radiation-only (R) and combination group (EY-L + R). Radiation was administered at 2.9 Gy/min in an XRAD-SmART instrument (225 kV, 13 mA). Additionally, a separate group of mice designated as R(early), received two doses of focused 10 Gy radiation on days 5 and 12. This was done to ensure that their average tumor volume was equal to that of the EY-L + R group at the time of the first radiation dose. Treatment was stopped after three weeks, and tumor growth was monitored for another 3 weeks. Given the 3 week-long washout period in this experiment, we refrained from using the tumor tissues from this experiment for immunohistochemistry (IHC). Instead, we conducted a similar experiment with another group of tumor-bearing mice and concluded it after 21 days (i.e., two days following the final radiation dose) followed by tumor harvest for IHC. Here, the radiation treatments were performed on the same days (i.e., days 12 and 19) in both combination and radiation-only groups to keep the interval the same between radiation and harvesting of tumors in these two groups. We injected 5 × 10^6^ cells per mouse in this experiment to reach an average tumor volume of 25 mm^3^ for starting the EY-L treatment faster (15 days post-inoculation) due to time constraint.

Similar experiments were conducted using subcutaneous Renca tumors developed in syngeneic Balb/c mice (1 × 10^6^ cells mouse). Here, we only kept the R(Early) group for the radiation-only treatment group for a more stringent comparison of the combination group with the radiation-only group. EY-L treatment started 2 weeks after inoculation at an average tumor volume of ~ 40 mm^3^ and discontinued after three weeks of treatment. Tumor growth was closely monitored until an IACUC-approved endpoint was reached for each mouse. As above, we also conducted a similar experiment with another group of tumor-bearing mice and concluded it after 21 days (i.e., two days following the final radiation dose) to harvest tumors for IHC analysis including any potential alterations in immune-cell infiltrations within the tumor microenvironment resulting from the treatment. EY-L treatment started 2 weeks after inoculation at an average tumor volume of ~ 30 mm^3^. Here, the radiation treatments were performed on the same days (i.e., days 12 and 19) in both combination and radiation-only groups to keep the interval the same between radiation and harvesting of tumors in these two groups.

### Immunohistochemistry

Tumors and spleens were harvested and fixed in neutral buffered 10% formalin at room temperature for 24 h. Then they were embedded in paraffin and 5 μm thick sections were cut for preparing slides. Hematoxylin and eosin (H&E), Ki67 (1:1000), phospho-p70S6K (1:200), survivin (1:400), CD45 (1:100), CD3 (1:100), CD4 (1:200), and CD8 (1: 1000) staining were performed in deparaffinized slides as applicable following the manufacturer’s instructions (DAB 150; Millipore). Slides were stained with stable diaminobenzidine and counterstained with hematoxylin. Finally, slides were digitized using an Aperio AT2 slide scanner (Leica) and analyzed using ImageScope software (Leica). A total of 30 visual fields (10 fields 0.25 mm^2^ each from 3 different tumor sections) were analyzed for quantification.

### Statistical analyses

Microsoft Excel (v 2312, part of Microsoft Office 365 package) and GraphPad Prism (v 9.4.0) were used for data analyses. One-way ANOVA followed by Tukey’s post-hoc analysis or double-sided unpaired two-tailed t-test was utilized to determine the probability of significant differences between treatment groups where applicable. For tumor growth curves, the endpoint or same-day tumor volumes were compared for statistically significant differences among each other using a double-sided unpaired two-tailed t-test where applicable. Statistical significance was defined as *p* < 0.05 (*), *p* < 0.01 (**), *p* < 0.001 (***), and *p* < 0.0001 (****) respectively. Error bars are indicative of calculated SD values.

## Results

### EY-L is a homogeneous, positively charged nanoformulation

The amount of lipid and drug components of the drug-loaded liposomes (E-L, Y-L, and EY-L) are reported in Supplementary Table S1 along with drug loading efficiency (DLE) and encapsulation efficiency (EE) values. The initial amounts of Everolimus and YM155 used during the preparation of liposomes were 0.4 mg and 0.8 mg per 1 mL of liposomes respectively. Everolimus, being a highly water-insoluble lipophilic drug, displayed an EE of 98.19% ± 2.13% in E-L and 96.73% ± 2.01% in EY-L due to its nearly complete incorporation in the liposome bilayer. YM155 displayed only 37.14% ± 1.70% EE in Y-L and 36.05% ± 2.35% in EY-L due to its hydrophilic nature. The DLE values for Everolimus in E-L and YM155 in Y-L were 7.42% ± 0.16% and 5.71% ± 0.26% respectively. On the other hand, The DLE values for Everolimus and YM155 in EY-L were 6.94% ± 0.14% and 5.17% ± 0.34% respectively. The EE values in dual drug-loaded liposomes (EY-L) did not show statistically significant alterations from the single drug-loaded ones, albeit they were slightly lower. Plausibly, the distinct spatial distribution of Everolimus and YM155 inside the liposomes is not affecting their individual encapsulation efficiencies. However, the DLE values of the EY-L differed more from E-L or Y-L due to the increased total weight of the EY-L liposomes containing both drugs over E-L or Y-L liposomes containing a single drug.

The average hydrodynamic size, polydispersity index (PDI), and zeta potential of E-L, Y-L, and EY-L are consolidated in Supplementary Table S2. The hydrodynamic diameters of E-L, Y-L, and EY-L were 62.15 nm ± 0.40 nm, 67.55 nm ± 0.24 nm, and 67.15 nm ± 0.31 nm, respectively. All the liposomal formulations had an average size of less than 100 nm which is suitable for better penetration through the tumor microenvironment [47]. The polydispersity indices of E-L, Y-L, and EY-L were 0.178 ± 0.015, 0.195 ± 0.007, and 0.205 ± 0.01, respectively, suggesting excellent uniformity of the liposomes. The zeta potentials of E-L, Y-L, and EY-L were 10.23 mV ± 2.4 mV, 32.7 mV ± 4 mV, and 37.5 mV ± 3.3 mV, respectively. A positive zeta potential indicates the stability of the liposomal suspension as well as stronger interaction with negatively charged cell membranes. All these liposomes were positively charged suggesting these formulations to be stable and efficient in cellular uptake [48].

### EY-L shows a robust antiproliferative effect in RCC cells in vitro

**Fig. 1:**
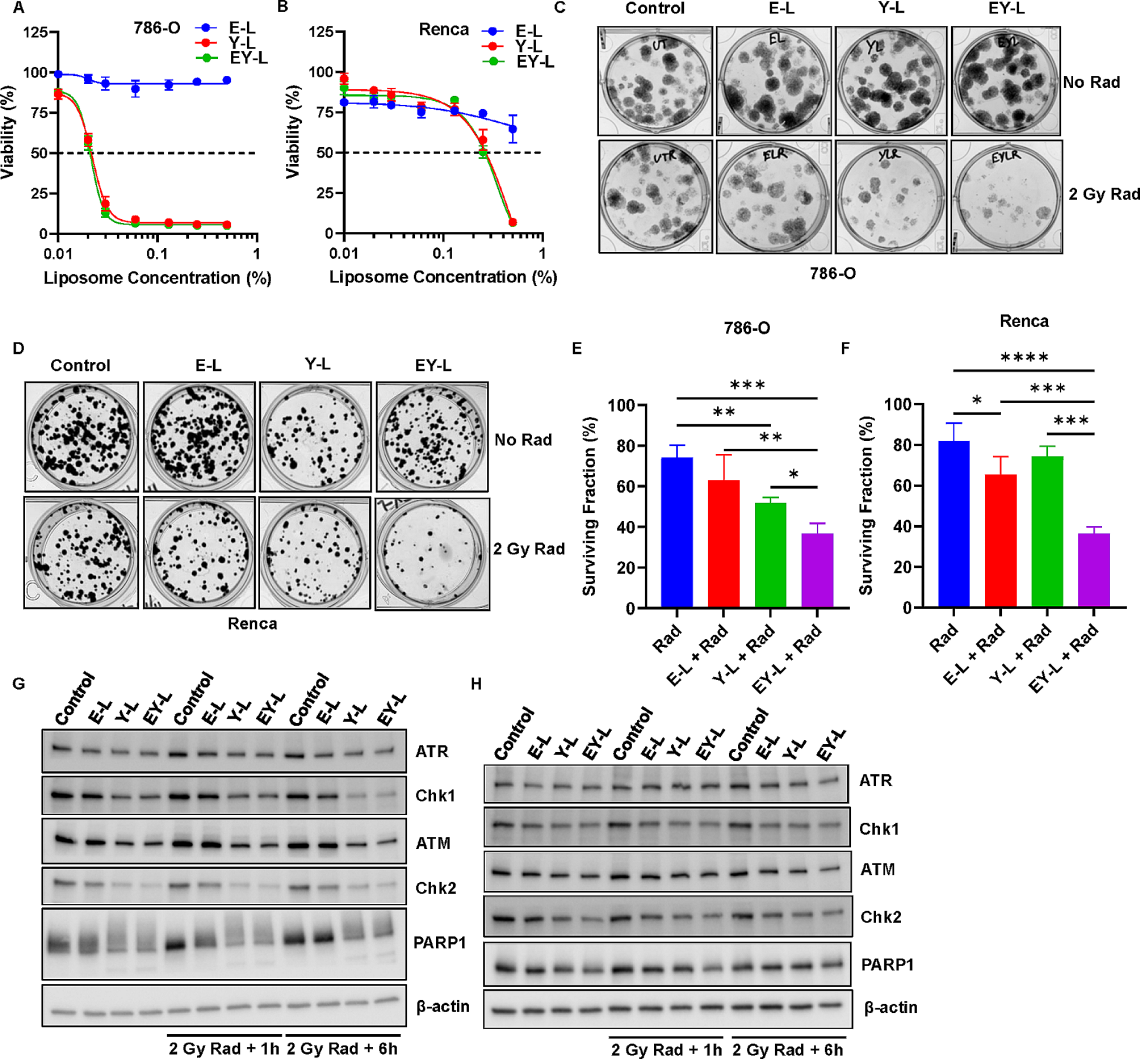
EY-L demonstrates antiproliferative effect and radiosensitization in RCC cells in vitro. MTS assay in 786-O (**A**) and Renca (**B**) cells treated with increasing concentrations of E-L, Y-L, or EY-L for 72 h (*n* = 4 wells per treatment condition). Clonogenic assay in 786-O (**C**) and Renca (**D**) cells for determining radiosensitization in vitro. Representative images of the colonies were included. Colonies greater than 50 cells were counted under a microscope and surviving fractions were plotted for 786-O (**E**) and Renca (**F**) cells. * *p* < 0.05, ** *p* < 0.01, *** *p* < 0.001, **** *p* < 0.0001. Western blot analysis of various DNA damage repair proteins from lysates of 786-O (**G**) and Renca (**H**) cells treated with sub-IC50 concentrations of E-L, Y-L, and EY-L for 48 h followed by exposure to 2 Gy radiation and incubation of 1 h and 6 h. A ‘no radiation’ control was included for each of the treatment groups

Following characterization, we assessed the in vitro cytotoxicities of the drug-loaded liposomal formulations in 786-O and Renca cells. Interestingly, E-L was not as cytotoxic as Y-L or EY-L although it did show 20-40% reduction in cell viability at the concentrations tested (Fig. 1A-B). Y-L and EY-L showed similar cytotoxicity, but 786-O cells were more sensitive towards Y-L or EY-L treatment than Renca, the IC50 values being more than tenfold less in 786-O cells (IC50 ~ 0.022% liposome) than in Renca cells (IC50 ~ 0.3% liposome). Here, 1% liposome is equivalent to ~ 4.1 µM (in E-L) or ~ 4.04 µM (in EY-L) everolimus, and ~ 6.7 µM (in Y-L) or ~ 6.51 µM (in EY-L) YM155. The IC50 values of cabozantinib and sunitinib were > 20 µM and ~ 5 µM in 786-O cells, and ~ 18 µM and ~ 7.5 µM in Renca cells, respectively(Supplementary Fig. S1). They are comparatively higher than the IC50 values of EY-L in 786-O (~ 0.022% EY-L, equivalent to ~ 88.88 nM E and ~ 143.22 nM Y) and Renca (~ 0.3% EY-L, equivalent to ~ 1.212 µM E and ~ 1.953 µM Y).

### EY-L sensitizes RCC cells toward radiation in vitro

Since both E and Y individually had been shown to increase the sensitivity of different cancer cells toward radiation, we investigated if there is any synergistic effect of EY-L in the radiosensitization of RCC cells in vitro over E-L or Y-L by performing colony formation assay. We used both 786-O and Renca cells in this experiment. 786-O cells formed dispersed-type colonies with diffused staining (Fig. 1C), whereas Renca cells formed well-defined colonies with good staining (Fig. 1D**)**. Nonetheless, the EY-L treated group led to the lowest surviving fraction post-radiation than the other treatment groups including the control, E-L, or Y-L (Fig. 1E-F). The Bliss combination indices for the radiosensitization of EY-L over E-L and Y-L were 0.809 and 0.503 for 786-O and Renca, respectively, suggesting a moderate-to-strong synergistic effect of the combination therapy (Supplementary Table S3).

Notably, we used sub-IC50 concentrations for the in vitro radiosensitivity assay (0.01% for 786-O and 0.125% for Renca) to show that even a sub-IC50 concentration of EY-L was enough to impart radiosensitivity in RCC cells. This is important and relevant since not all cells in the tumor get effective concentrations of the drugs to be eliminated. Our strategy has potentially dual benefits for tumor growth inhibition in vivo. The cells getting effective concentrations will be eliminated by the drugs’ effect alone. However, cells receiving lower amounts may still be sensitized to radiation and will be eliminated following radiation therapy.

### EY-L demonstrates superior inhibition of mTOR and survivin over E-L and Y-L, respectively

Western blot experiments demonstrate that EY-L was superior to E-L and Y-L in inhibiting phosphorylation of p70S6K (downstream of mTOR) and survivin expression, respectively (Supplementary Fig. S2). This suggests that Everolimus and YM155 act synergistically to augment each other’s function when combined in a single formulation. Interestingly, the same amount of Everolimus alone (as E-L) was not able to inhibit phosphorylations of p70S6K in any of the cells. The nominal observable changes in phenotype, such as minor reductions in proliferation, colony formation, or expression of DNA damage response markers following E-L treatment as depicted in Fig. 1, are consistent with the lack of E-L ‘s efficacy in suppressing mTOR signaling at these lower doses. However, higher concentrations of E-L could still effectively inhibit the phosphorylations of mTOR and p70S6K indicating the on-target effect of Everolimus is intact in E-L (Supplementary Fig. S3). Similarly, Y-L was effective at reducing survivin expression in 786-O cells only, but not in Renca cells. In contrast, EY-L was equally effective in inhibiting p70S6K phosphorylation and survivin expression in both cell lines. While this may seem perplexing, we were using sub-IC50 values (0.01% for 786-O, 0.125% for Renca) which were used in radiosensitization studies above, so it is possible that individual drugs are not effective at these lower concentrations. However, our goal in this experiment was to show that even at these lower concentrations, the combination is better in inhibiting both mTOR and survivin pathways synergistically, which is reflected in the observed data. Notably, YM155 had previously been shown to inhibit the phosphorylation of mTOR and S6 in several cancer types [49, 50]. Similarly, everolimus or temsirolimus was shown to inhibit survivin levels as well [26, 51]. In addition, the combination of temsirolimus and YM155 had been shown to inhibit survivin synergistically [26]. Hence, this synergistic action might be the reason that EY-L effectively inhibited both pathways at these lower concentrations, where the individual drugs exhibited diminished efficacy or no efficacy at all. Additionally, since EY-L inhibits both phospho-p70S6K and survivin as shown in Supplementary Fig. S1, it is implied that the enhanced phenotypic outcomes associated with EY-L likely stem from the synergistic combination of on-target effects of everolimus and YM155.

### EY-L inhibits multiple DNA damage repair mechanisms

Efficient DNA damage repair mechanisms are required to alleviate the harmful effects of radiation. These pathways are typically exploited by various cancer cells to maintain their radioresistant nature. Some of the crucial proteins involved in DNA damage repair include PARP1 (widely recognized as a first-line responder molecule in DNA damage response), ATM/Chk2 (double-stranded break repair), and ATR/Chk1 (single-stranded break repair). Not surprisingly, EY-L was highly effective and in most cases was better than E-L or Y-L in reducing the expressions of these proteins, even subduing any increase post-radiation in some instances (Fig. 1G-H).

### EY-L demonstrates a strong antitumor effect in a subcutaneous syngeneic murine RCC model

**Fig. 2:**
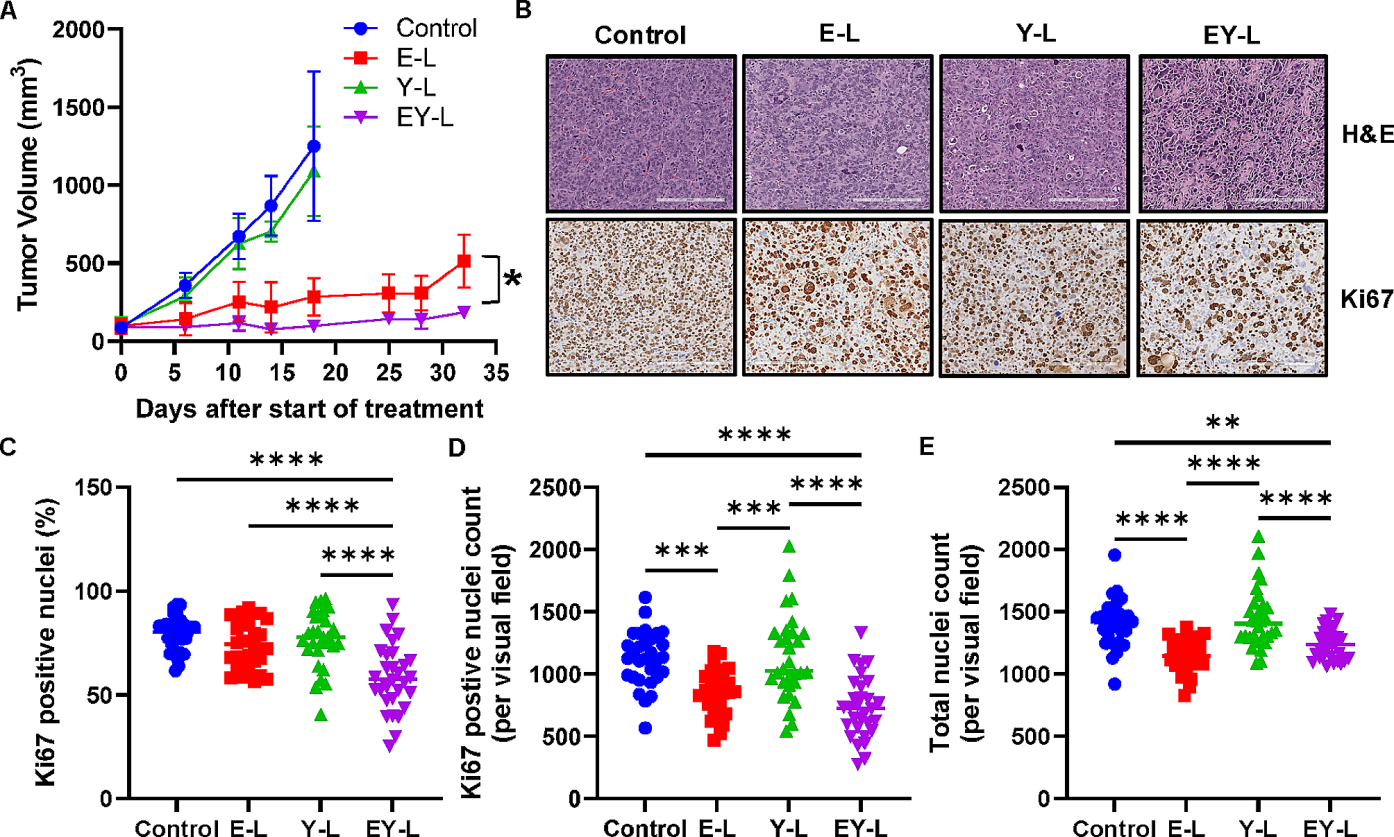
EY-L demonstrates a remarkable antitumor effect in a subcutaneous syngeneic mouse model of RCC. (**A**) Growth curves for subcutaneous Renca tumors treated with E-L, Y-L, and EY-L (*n* = 5 mice per group). (**B**) Representative images of H&E and Ki67 stained tumor sections from the above experiment. Bar length = 200 μm. Quantitation of percentage of Ki67-positive nuclei (**C**), Ki67-positive nuclei count (**D**), and total nuclei count (**E**) in tumor sections (*n* = 30, 10 visual fields 0.25 mm^2^ each from 3 different tumor sections per group). * *p* < 0.05, ** *p* < 0.01, *** *p* < 0.001, **** *p* < 0.0001

Inspired by the superior in vitro efficacy of EY-L, we proceeded to analyze the in vivo efficacy of the drug-loaded liposomes in a highly aggressive syngeneic mouse RCC model developed by subcutaneous implantation of Renca cells in immune-competent Balb/c mice. Anti-PD-1 therapy in this model led to a modest ~ 48% tumor growth inhibition (Supplementary Fig. S4). Both E-L and EY-L displayed remarkable tumor growth inhibition throughout the study (~ 77% and ~ 92%, respectively, compared with control on Day 18), EY-L being the most effective treatment group (Fig. 2A). The individual tumor growth curves from this experiment are provided in Supplementary Fig. S5. Interestingly, YM-155 did not show any visible tumor growth inhibition as a single liposomal formulation (Y-L) in this experiment but augmented the efficacy of everolimus when combined in the same liposomal formulation (EY-L). In contrast, E-L was not as cytotoxic as Y-L or EY-L but showed significant tumor growth inhibition. These results suggest that the in vitro and in vivo efficacy of drugs may not always reflect each other [52]. For in vitro experiments, the therapeutic agents are present in the immediate vicinity of the cancer cells, but they need to cross several barriers to reach the tumor cells in vivo. The pharmacokinetics, biodistribution, and clearance of the drugs play a major role in their therapeutic efficacy in vivo. Moreover, drugs like everolimus not only affect the tumor cells directly but also interact with the other cells present in the tumor microenvironment, which may contribute to its antitumor effect [53].

The H&E and Ki67 staining of the tumor sections demonstrates strong antiproliferative activity in EY-L-treated tumors (Fig. 2B-C). Interestingly, E-L treated tumors did not show any significant change in the percentage of Ki67-positive nuclei although E-L caused significant tumor growth inhibition. This prompted us to perform a careful reexamination of the Ki67-stained tissues, where we observed that the Ki67-positive nuclei per visual field (approximately 0.25 mm^2^ per field, 30 fields per treatment group) are significantly less in E-L-treated tumors than in control tumors (Fig. 2D). However, the total nuclei count also decreased significantly in the E-L-treated group (Fig. 2E), thus increasing the percentage of Ki67-positive nuclei. Hence, we did not observe a significant difference in the percentage of Ki67-positive nuclei between control and E-L-treated tumors. Nonetheless, the decrease in Ki67-positive as well as total nuclei count per visual field in EY-L treated tumors indicate a significant antiproliferative effect of E-L, which explains its remarkable antitumor effect. In addition, assessment of phospho-p70S6K expression in these tumor sections showed that both E-L and EY-L led to significant reductions in the p70S6K phosphorylation in vivo (Supplementary Fig. S6). Similarly, both Y-L and EY-L showed reduced the percentage of survivin positive nuclei. However, EY-L demonstrated superior inhibition in both markers. These findings provide additional evidence of the on-target effects of E-L, Y-L, and EY-L in vivo.

Interestingly, none of the treatments significantly changed the percentage of tumor-infiltrating CD4 + T cells, but EY-L showed a slight increase (Supplementary Fig. S7). On the other hand, EY-L showed a statistically significant increase in CD8 + T cell infiltration from control and E-L, but not Y-L. The spleens of the treated mice did not show any significant changes in the percentage of CD4 + or CD8 + T-cells in their T-cell zones (Supplementary Fig. S8). Collectively, these results suggests that EY-L affects tumor proliferation more than the immune system.

### EY-L impedes tumor growth in an orthotopic syngeneic murine RCC model

**Fig. 3:**
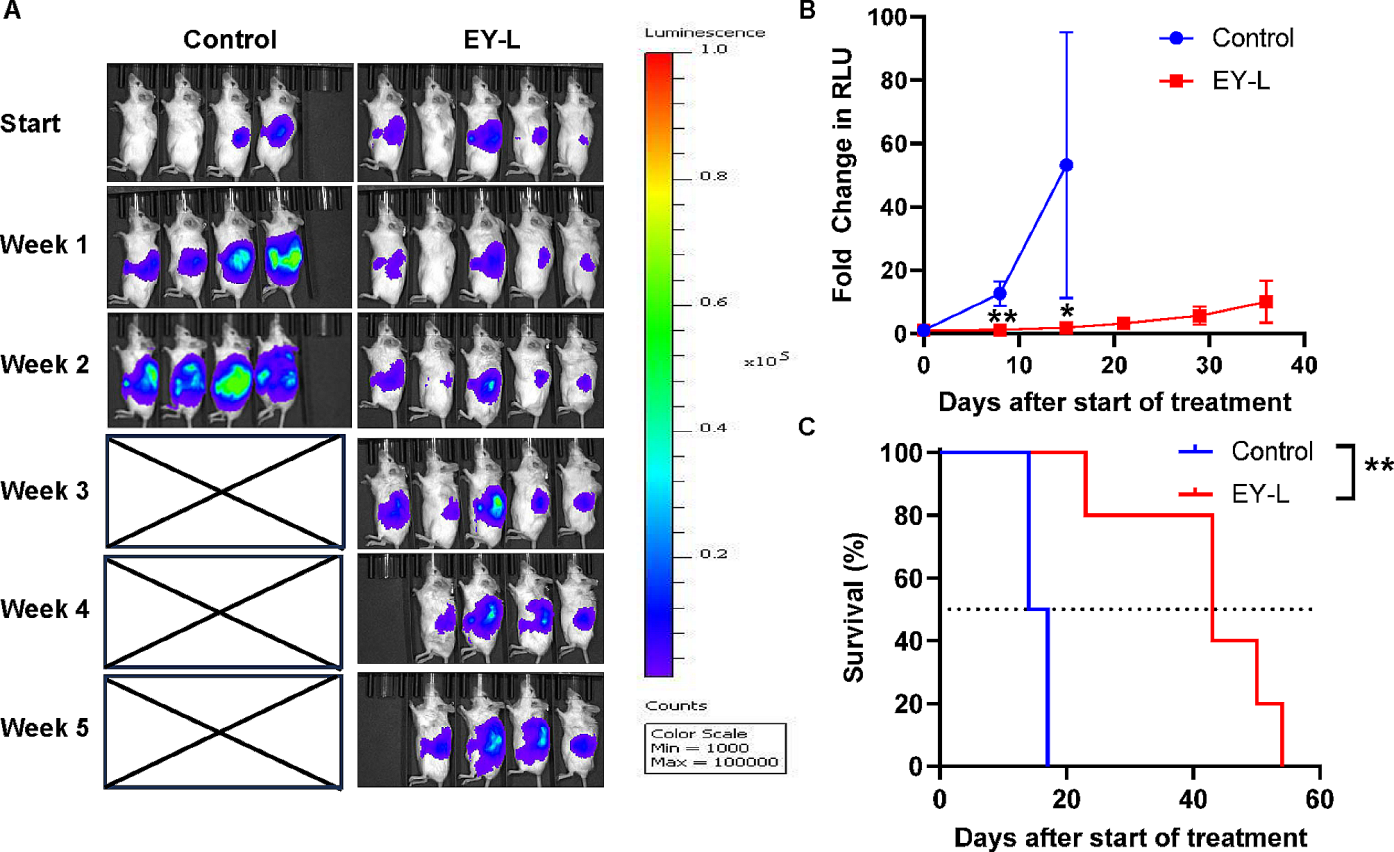
EY-L demonstrates a remarkable antitumor effect in an orthotopic syngeneic mouse model of RCC. (**A**) Bioluminescence images for orthtotopic Renca tumors treated with E-L, Y-L, and EY-L (*n* = 4 for the control group, *n* = 5 for EY-L group). The control mice reached the endpoint due to aggressive tumor growth after 2 weeks of starting treatment. (**B**) Tumor growth curves plotted as a fold change in RLU from initial measurements. (**C**) Median overall survival from the above experiment. * *p* < 0.05, ** *p* < 0.01

We further tested the efficacy of EY-L in an orthotopic syngeneic mouse ccRCC model developed by subcapsular implantation of luciferase-labeled Renca cells in immune-competent Balb/c mice. Since EY-L was the most effective in the previous experiment, we did not include E-L or Y-L in this experiment or further in vivo experiments. EY-L showed significant tumor growth inhibition (Fig. 3A-B) and enhanced median survival (43 days vs. 15 days in the control group) (Fig. 3C) compared to the control group in this model. The individual tumor growth curves from this experiment are provided in Supplementary Fig. S9.

### EY-L sensitizes RCC xenograft tumors toward radiation in vivo

**Fig. 4:**
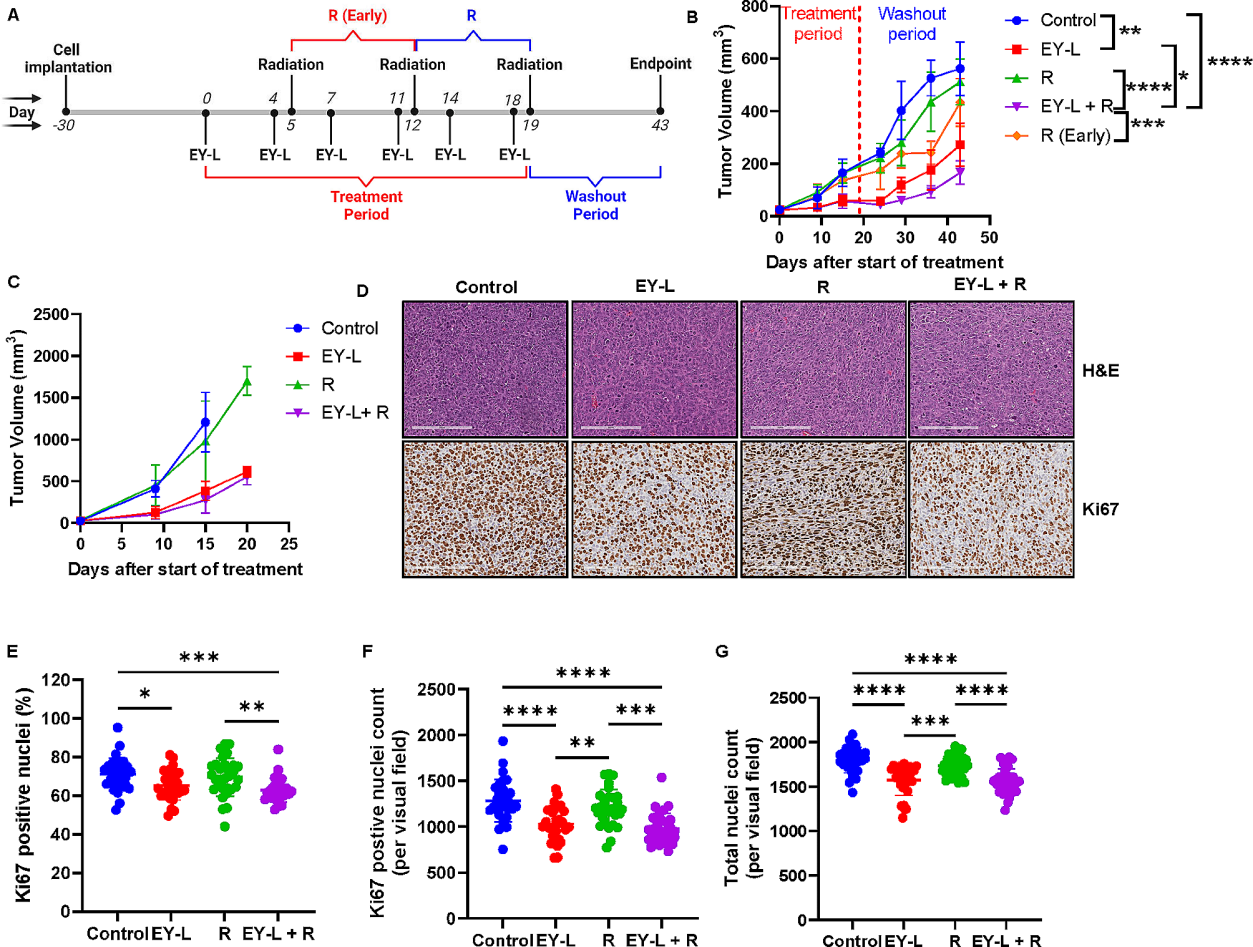
EY-L augments radiation therapy in an RCC xenograft model. (**A**) Timeline of the experiment. (**B**) Growth curves of subcutaneous 786-O tumors treated with EY-L, Radiation, and their combination (*n* = 5). Untreated control and R(Early) groups were also included for comparison. (**C**) A similar experiment was performed but was stopped 2 days after the final dose of radiation to harvest the tumors for immunohistochemistry. Here, only the R group was included to keep the washout period the same between treatments. The tumor growth curves were steeper here due to inoculation of a higher number of cells. (**D**) Representative images of H&E and Ki67 stained tumor sections from the above experiment. Bar length = 200 μm. Quantitation of percentage of Ki67-positive nuclei (**E**), Ki67-positive nuclei count (**F**), and total nuclei count (**G**) in tumor sections (*n* = 30, 10 visual fields 0.25 mm^2^ each from 3 different tumor sections per group). * *p* < 0.05, ** *p* < 0.01, *** *p* < 0.001, **** *p* < 0.0001

Inspired by the observed results from the in vitro radiosensitivity experiments and Western Blot analysis, we evaluated the in vivo radiosensitivity of EY-L. We first used subcutaneous 786-O xenografts developed in SCID mice to evaluate the in vivo radiosensitization potential of EY-L in the absence of any additional effects due to the immune system. We evaluated only EY-L in this experiment since it was superior to E-L and Y-L in vitro. The experiment timeline is provided in Fig. 4A. Treatment was stopped after three weeks (treatment period), and tumor growth monitoring was continued for another 3 weeks of washout period. Since, EY-L treatment was expected to impede the tumor growth compared to the control group, the tumor volume of radiation only (R) group would be higher than EY-L + R group ay Day 12, when the first dose of radiation would be administered. Hence, we also included another group, R(Early), where radiation doses were given one week earlier (Day 5 and Day 12) to match with the tumor volume of EY-L + R group during the administration of first dose of radiation. Indeed, on Day 12 at the time of 1st radiation exposure, the tumor volume of EY-L (or EY-L + R since they are the same until this point) was lower than the control (or R since they are the same until this point) (Fig. 4B). However, as can be seen from Supplementary Fig. S10, the Day 5 tumor volume of the R(Early) group (~ 50 mm^3^) is equal to the Day 12 tumor volume of the EY-L + R group (~ 50 mm^3^). Interestingly, R (Early) group showed an initial difference in tumor growth from the R group due to early exposure to radiation but after 6 weeks there was no significant difference between them (Fig. 4B). EY-L + R group showed significant impedance in tumor growth compared to all other groups including EY-L, suggesting the augmentation of radiation therapy by EY-L. The individual tumor growth curves from this experiment are provided in Supplementary Fig. S11. The body weights of mice from all the above treatment groups including EY-L + R did not show significant changes suggesting no treatment-induced toxicity (Supplementary Fig. S12).

We performed immunohistochemistry from the Formalin-fixed paraffin-embedded (FFPE) tumor tissues obtained from a similar experiment that was terminated 2 days after the final dose of radiation to avoid any loss of treatment-induced alteration in tumor sections due to 21-day long washout period. Interestingly, the tumor growth curves were steeper in this experiment since we inoculated 5 × 10^6^ cells per mouse (Fig. 4C and Supplementary Fig. S13), but the growth curve trends were similar to the previous experiment. Nonetheless, both EY-L and EY-L + R groups showed significant reductions in the percentage of Ki67 positive nuclei, Ki67-positive nuclei count, and total nuclei count compared to control, but EY-L + R group was slightly better (Fig. 4D-G).

### EY-L sensitizes syngeneic RCC tumors toward radiation in vivo

**Fig. 5:**
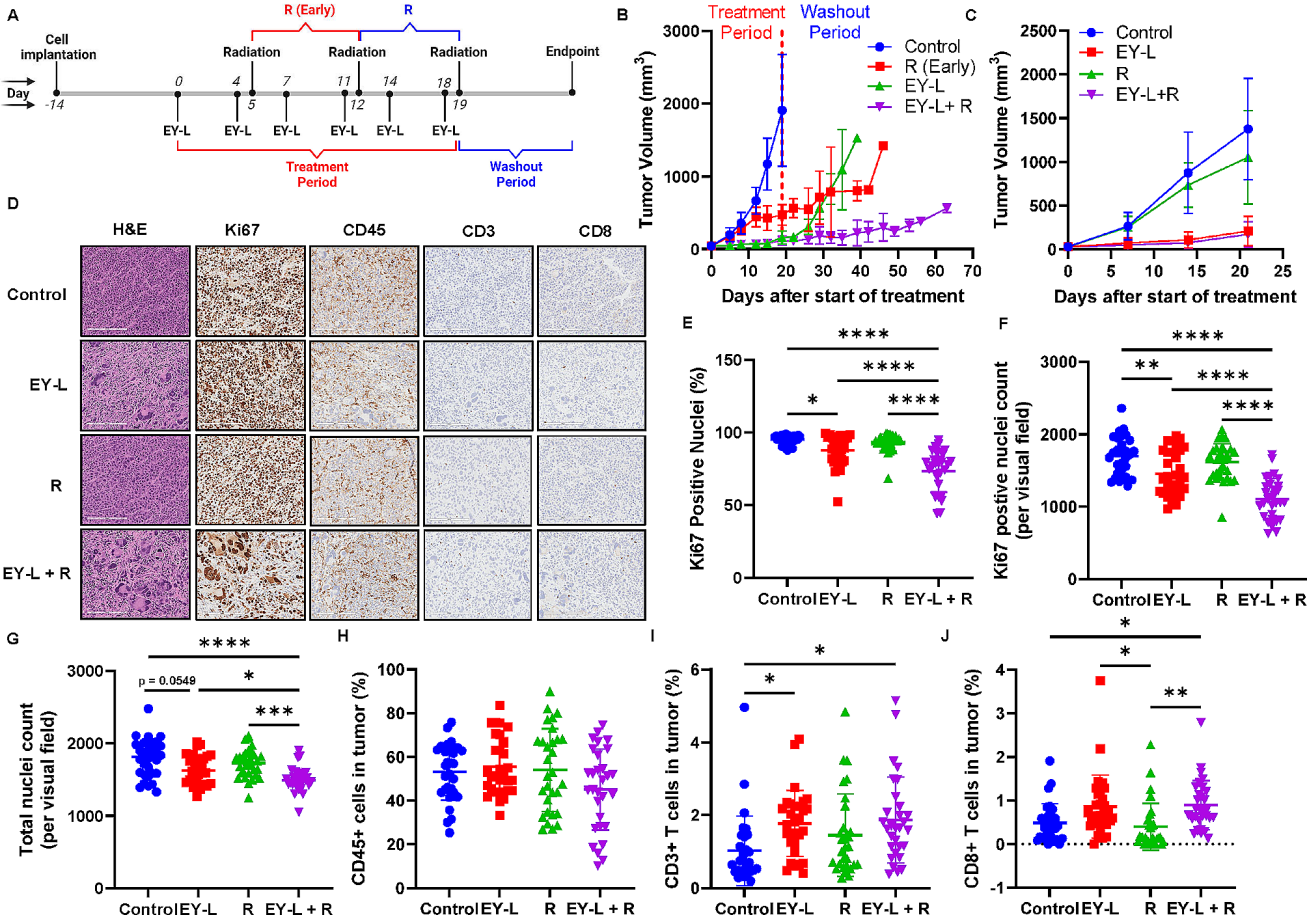
EY-L augments radiation therapy in a murine syngeneic RCC model. (**A**) Timeline of the experiment. (**B**) Growth curves of subcutaneous Renca tumors treated with EY-L, and its combination with radiation. Untreated control and R(Early) groups were also included for comparison. No statistics was performed due to different endpoints. (**C**) A similar experiment was performed but was stopped 2 days after the final dose of radiation to harvest the tumors for immunohistochemistry. Here, the R (Early) group was replaced with the regular R group to keep the washout period the same between treatments. (**D**) Representative images of H&E, Ki67, CD45, CD3, and CD8 stained tumor sections from the above experiment. Bar length = 200 μm. Quantitation of percentage of Ki67-positive nuclei (**E**), Ki67-positive nuclei count (**F**), total nuclei count (**G**), CD45 + cells (**H**), CD3 + cells (**I**), and CD8 + cell (**J**) in tumor sections (*n* = 30, 10 visual fields 0.25 mm^2^ each from 3 different tumor sections per group). * *p* < 0.05, ** *p* < 0.01, *** *p* < 0.001, **** *p* < 0.0001

A similar experiment was performed in subcutaneous Renca tumors developed in syngeneic Balb/c mice to assess if the immune system plays any additional role in EY-L mediated radiosensitization. Here, we only kept the R (Early) group for the radiation-only treatment group for a more stringent comparison of the efficacy of the combination group with the radiation-only group. A similar experimental timeline was followed (Fig. 5A). Treatment was stopped after 3 weeks, and tumor growth was monitored until an IACUC-approved endpoint was reached for each mouse. As anticipated, EY-L + R treatment led to a noticeable inhibition of tumor progression compared to the control, EY-L, or R (Early) groups (Fig. 5B). The individual tumor growth curves from this experiment are provided in Supplementary Fig. S14. The body weights of mice from EY-L and EY-L + R groups did not show significant changes suggesting no treatment-induced toxicity, but both control and R(Early) groups showed greater than 10% weight loss (Supplementary Fig. S15).

Similar to the 786-O xenograft experiment, we repeated the experiment in another set of tumor-bearing mice and stopped the experiment after 21 days (i.e., 2 days after the final radiation dose) to harvest tumors for immunohistochemistry including any alterations in immune-cell infiltrations in the tumor microenvironment due to treatment (Fig. 5C). The radiation dosing schedules were kept same between R and EY-L + R (Day 12 and Day 19) in this experiment to remove any disparity in treatment-induced alterations in endpoint immunohistochemistry due to different dosing schedules and washout periods. The individual tumor growth curves from this experiment are provided in Supplementary Fig. S16. Immunohistochemistry was performed on FFPE tumor tissue sections for H&E, Ki67, CD45, CD3, and CD8 (Fig. 5D). The quantification of Ki67, CD45, CD3, and CD8 staining was performed as well (Fig. 5E-H). The EY-L + R group showed significant reductions in the percentage of Ki67 positive nuclei (Fig. 5E), Ki67 positive nuclei count (Fig. 5F), and total nuclei count (Fig. 5G) among all the groups. CD45 staining was not significantly affected among the treatment groups, although the EY-L + R group showed slightly lower abundance (Fig. 5H). CD3 + T cells were significantly higher in both EY-L and EY-L + R treatment groups compared to the control group (Fig. 5I). Interestingly, CD8 + T cells in both EY-L and EY-L + R treatment groups were significantly higher than control or R groups (Fig. 5J). However, no significant difference was observed between the EY-L and EY-L + R groups. Nonetheless, this experiment suggests an additional effect of the immune system in EY-L mediated radiosensitization of the Renca tumors.

### EY-L induced mitotic catastrophe in RCC tumors which is aggravated by radiation exposure

**Fig. 6:**
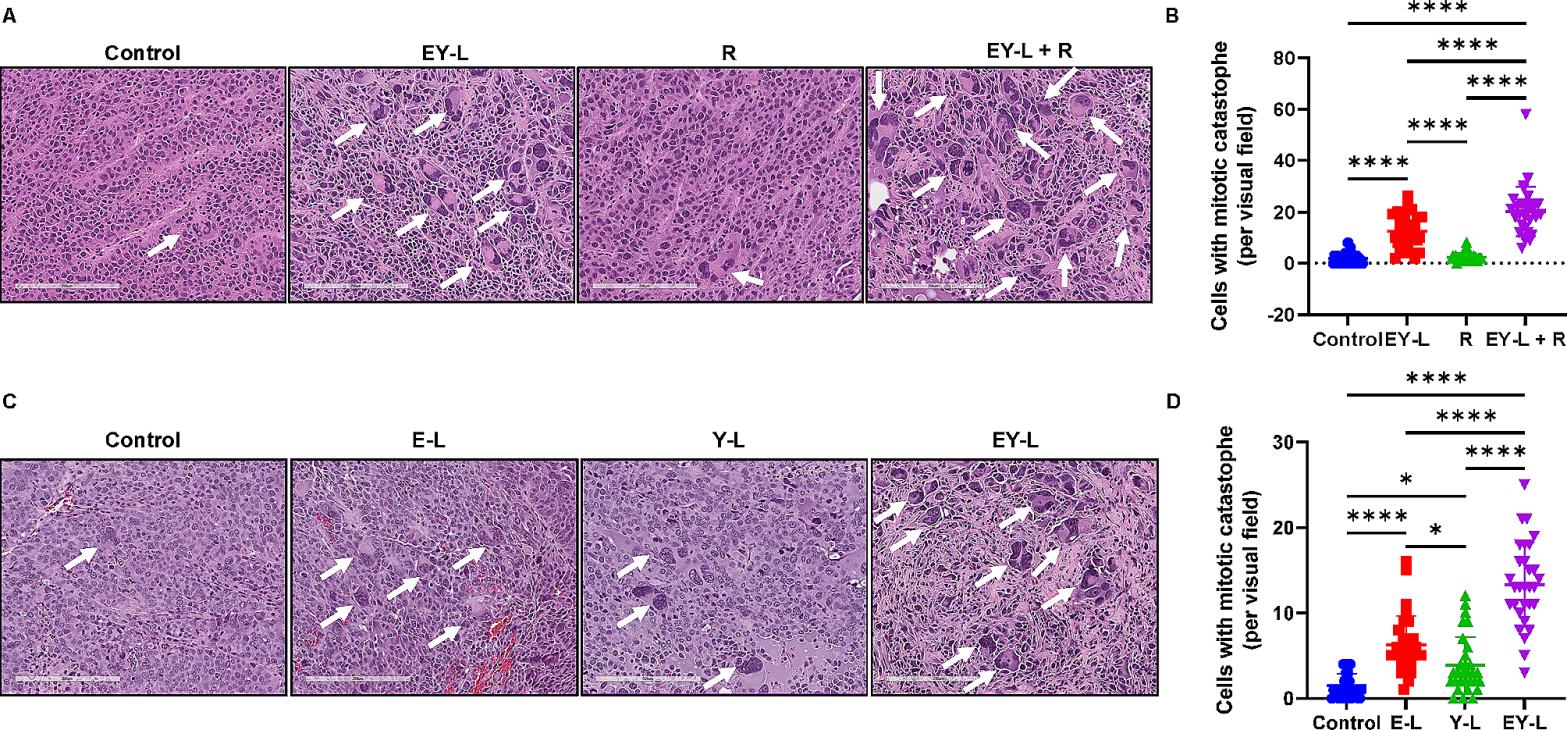
EY-L induces mitotic catastrophe in RCC tumors which is further enhanced by radiation therapy. Representative images of H&E-stained tumor sections from experiments shown in Fig. 5 (**A**) and Fig. 2 (**C**) showing the presence of large multinucleated cells (indicated by white arrows) as evidence of mitotic catastrophe. (**B**, **D**) Quantification of mitotic catastrophe in these tumor sections (*n* = 30, 10 visual fields 0.25 mm^2^ each from 3 different tumor sections per group). * *p* < 0.05, **** *p* < 0.0001

The H&E staining of the tumor tissue sections in Fig. 5D showed the presence of several multinucleated cells in the EY-L and EY-L + R treated tumors, the abundance being higher in the combination group. Giant multinucleated cells characterized by missegregated and uncondensed chromosomes are the morphological markers of mitotic catastrophe, and this has previously been used for quantifying mitotic catastrophe in tumor Sect. [54]. Therefore, this prompted us to analyze the extent of mitotic catastrophe in these tumors by counting the cells with deformed or multisegmented nuclei in H&E-stained tumor sections (Fig. 6A). Interestingly, the quantification of cells with deformed or multinucleated nuclei showed a significant increase in both EY-L and EY-L + R treated tumors than control or radiation-treated tumors, (Fig. 6B). However, the counts were significantly higher in EY-L + R than in EY-L, suggesting a significantly higher incidence of mitotic catastrophe in the EY-L + R group. We also went back and checked if we could see similar induction of mitotic catastrophe in the in vivo experiment comparing E-L, Y-L, and EY-L in the Renca tumors shown in Fig. 2A. As expected, we observed a noticeable induction of mitotic catastrophe as illustrated by the presence of cells with deformed nuclei in the EY-L treated group and to some extent in the E-L and Y-L treated groups (Fig. 6C-D). Although survivin inhibition has previously been implicated in mitotic catastrophe, to our knowledge this is the first report showing induction of mitotic catastrophe by everolimus (as E-L).

To confirm whether EY-L mediated mitotic catastrophe is present in the immunodeficient model, we analyzed H&E-stained 786-O tumor sections from the experiment shown in Fig. 4C. We observed that EY-L-mediated mitotic catastrophe was present in 786-O tumors as well but to a lesser extent than in Renca tumors (Supplementary Fig. S17). This indicates a plausible augmenting role of the immune system in EY-L-mediated mitotic catastrophe which is reflected by more prominent and long-lasting inhibition of tumor growth in the immunocompetent model.

## Discussion

The primary objective of RT in radiation oncology is to hinder the proliferation of cancer cells and ultimately eliminate them. RT employs various mechanisms to achieve this, including apoptosis, autophagy, mitotic death (or mitotic catastrophe), necrosis, and senescence [55]. However, given that radiation can harm both cancerous and healthy cells, the focus of RT is to maximize the radiation dose directed at the tumor while minimizing exposure to adjacent normal cells or those in the path of the radiation. Advanced technologies employed in RT delivery such as SBRT facilitate the administration of a maximum radiation dose to the tumor while sparing healthy tissues [14].

Another strategy to enhance radiation therapy treatment outcomes involves the use of radiosensitizers for radiosensitization of cancer cells [15]. Radiosensitization is a process aimed at heightening the vulnerability of cancer cells to radiation-induced damage, while simultaneously minimizing potential harm to the adjacent healthy tissues. Radiosensitizers can affect cancer cells in various ways including increasing ROS within the cancer cells, inhibiting DNA repair mechanisms, modifying the tumor microenvironment, and targeting specific molecular pathways or proteins involved in cell survival and radiation resistance [56]. In recent years, there has been a substantial surge in interest regarding the use of radiosensitizers to augment the efficacy of radiotherapy. Radiosensitizers can be categorized into three main groups based on their composition: small molecules, macromolecules, and nanomaterials [57]. Radiosensitizers being evaluated in various clinical trials include Cisplatin, Gemcitabine, Olaparib, Paclitaxel, Temozolomide, Cetuximab, noble metal nanoparticles, and heavy metal nanoparticles [57].

We included everolimus and YM155, inhibitors of mTOR and survivin, respectively, as radiosensitizers in the present study. The selection of this combination was partly rationalized based on the findings of a previous study demonstrating that YM155 was able to overcome resistance to mTOR inhibitors in renal cancer [26]. The result obtained from the tumor growth inhibition study in a subcutaneous murine RCC model further corroborated these observations (Fig. 2A). EY-L was effective in impeding tumor growth and enhancing survival in orthotopic tumors as well (Fig. 3A-C).

Additionally, both mTOR and survivin are implicated in cell proliferation, survival, and DNA damage response pathways, which are responsible for imparting RT resistance in cancer [18, 33–35]. Consequently, both mTOR inhibitors and survivin inhibitors have gained significant attention in recent years due to their potential role as radiosensitizers in cancer treatment. Several clinical trials have explored the combination of mTOR inhibitors with radiation therapy in various cancer types [19–23]. These trials mostly aimed to assess the safety and efficacy of this combination strategy and findings from these studies suggest potential benefits. Based on the above observations, we hypothesized that simultaneously inhibiting these two pathways would augment the effect of radiation on cancer cells synergistically. Indeed, the clonogenic assay in our study showed a moderate-to-strong synergistic effect of this combination in two different RCC cell lines (Fig. 1C-F and Supplementary Table S3). The combination also efficiently reduced the expressions of multiple DNA damage response elements (Fig. 1G-H). Hence, it is not a surprise when the combination augmented the effects of radiation in a subcutaneous RCC xenograft model (Fig. 4B).

However, this xenograft model does not consider the effect of an intact immune system on the outcome of RT. RT not only exerts cytotoxic effects on tumor cells but also amplifies antitumor immunity by modifying the tumor microenvironment (TME) to elicit a potent antitumorigenic immune response [58–61]. RT induces immunogenic cell death, resulting in the release of various cytokines and chemokines into the TME, which serve as chemoattractants facilitating the infiltration of dendritic cells (DCs) to the tumor site [62]. The activation of DCs and the upregulation of cytotoxic T lymphocytes are believed to be the cause of the radiation-induced antitumorigenic immune response [63, 64]. Conversely, RT has demonstrated the ability to induce immunosuppression by promoting the infiltration of regulatory T cells (Tregs) and myeloid-derived suppressor cells (MDSCs) into the TME [65–67].

Everolimus, typically immunosuppressive, has been shown to increase the abundance of Tregs and MDSCs in both the TME and circulation [68]. Although the tumor-targeted liposomal formulation is anticipated to reduce the systemic exposure of everolimus, its potential to elevate immunosuppressive Tregs and MDSCs in the tumor microenvironment, thereby counteracting any immune-mediated enhancement of radiation therapy, cannot be disregarded. On the contrary, survivin, released from cancer cells into the TME, serves as a modulator of the T cell response, inhibiting their proliferation and inducing a shift to a type 2 response [69]. Therefore, the presence of the survivin inhibitor YM155 in EY-L is expected to mitigate the immunosuppressive effect of everolimus to some extent. Indeed, our data suggests that EY-L treatment, either alone or in combination with radiation, demonstrated slightly increased CD8 + T cell infiltration in Renca tumors (Fig. 5J), which may be responsible for a comparatively better antitumor response for EY-L + R treatment in Renca tumors than 786-O tumors (Fig. 5B). While the effectiveness of EY-L in immunodeficient mice implies that the immune system is not a prerequisite for EY-L-mediated radiosensitization, it does not necessarily mean that EY-L-mediated radiosensitization would not be boosted further by CD8 + T cell infiltration in Renca tumors. The increased presence of CD8 + T cells could work in tandem with EY-L’s intrinsic immune-system independent radiosensitization effect to enhance the antitumor response in immunocompetent models, as the role of tumor-infiltrating CD8 + T cells in enhancing response to radiotherapy is well established [70–72]. The sustained growth inhibition observed in the Renca model supports this as a plausible mechanism.

Mitotic catastrophe is considered a form of cell death that occurs during or after abnormal mitosis. It is an important aspect of the cellular response to DNA damage, including damage induced by radiation [55]. When this damage is severe and beyond repair, the cell may undergo mitotic catastrophe as a response. Typically, cells have mechanisms to halt the cell cycle to allow for repair in response to DNA damage. If the damage is extensive and irreparable, cells may be arrested in the G2 phase of the cell cycle. Despite the cell cycle arrest, some cells may attempt to undergo mitosis. This is problematic because the damaged DNA is often unevenly distributed between the daughter cells, leading to genomic instability. This can result in cell death or the generation of cells with abnormal chromosome numbers and structures. Mitotic catastrophe often triggers programmed cell death pathways, such as apoptosis or necrosis, as a protective mechanism to eliminate cells with severely damaged DNA and prevent the propagation of genetic abnormalities [73]. This has led cancer researchers across the globe to exploit mitotic catastrophe as an attractive avenue for cancer therapy [74].

Interestingly, survivin participates in the chromosomal passenger complex and ensures accurate separation of sister chromatids and microtubule stabilization at the late stages of mitosis [75]. Consequently, loss-of-function of the gene encoding survivin can lead to mitotic disturbances such as mitosis delay, chromosome displacement, and cell accumulation in prometaphase [76]. RNAi-based survivin knockdown has been previously shown to induce mitotic catastrophe in multiple cancer and non-cancer cell lines [54, 77–79]. Additionally, Y-L downregulates Chk1 and Chk2, both of which are negative regulators of mitotic catastrophe [80, 81]. On the other hand, mTOR inhibitors alone are not known to induce mitotic catastrophe but a few studies have shown that a combination of mTOR inhibitors with other genotoxic agents such as Chk1 inhibitor and HASPIN inhibitor were able to induce mitotic catastrophe in cancer cells [82, 83]. Since YM155 (as Y-L) inhibits Chk1 (Fig. 1G-H), it is plausible that a combination of everolimus with YM155 would do the same. Indeed, our data shows that EY-L, both alone and in combination with radiation, induced mitotic catastrophe in RCC tumors in vivo, as illustrated by the abundance of multinucleated cells in the H&E-stained tumor sections (Fig. 6A-B). However, we also show the induction of mitotic catastrophe by everolimus as E-L (Fig. 6C-D), which is a first-time report of such phenomenon to the best of our knowledge.

## Conclusion

In summary, our study utilized a rational combination of an mTOR inhibitor and a survivin inhibitor in a tumor-targeted liposomal formulation to augment radiation therapy in renal cancer by inhibiting DNA damage repair and enhancing mitotic catastrophe. The combination itself showed excellent tumor growth inhibition by synergistically blocking mTOR and survivin, so, the proposed strategy is poised to act through a two-pronged assault on tumors: (a) directly affecting tumor growth and (b) sensitizing tumors toward radiation. While the present study is focused on renal cancer, this strategy may also be useful in other cancer indications since both everolimus and YM155 have been shown to act as radiosensitizers in a variety of cancers including lung cancer, breast cancer, prostate cancer, and glioblastoma.

### Electronic supplementary material

Below is the link to the electronic supplementary material.

Supplementary Material 1

Supplementary Material 2

Supplementary Material 3

Supplementary Material 4

## Data Availability

All data generated or analyzed during this study are included in this published article and its supplementary information files.

## References

[1] Siegel, Miller, Wagle, Jemal A (2023). Cancer statistics, 2023. Cancer J Clin.

[2] Low G, Huang G, Fu W, Moloo Z, Girgis S. Review of renal cell carcinoma and its common subtypes in radiology. World J Radiol. 2016;8(5). 10.4329/wjr.v8.i5.484 PMC4882405 27247714

[3] Dudani S, de Velasco G, Wells JC, Gan CL, Donskov F, Porta C et al. Evaluation of Clear Cell, Papillary, and Chromophobe Renal Cell Carcinoma Metastasis Sites and Association with Survival. JAMA Netw Open. 2021;4(1). 10.1001/jamanetworkopen.2020.21869 PMC7821027 33475752

[4] Heng DYC (2016). The next 10 years: challenges for the future and overcoming resistance to targeted therapies for renal cell carcinoma. Can Urol Association J.

[5] Motzer RJ, Escudier B, McDermott DF, George S, Hammers HJ, Srinivas S (2015). Nivolumab versus Everolimus in Advanced Renal-Cell Carcinoma. N Engl J Med.

[6] Motzer RJ, Tannir NM, McDermott DF, Arén Frontera O, Melichar B, Choueiri TK (2018). Nivolumab plus Ipilimumab versus Sunitinib in Advanced Renal-Cell Carcinoma. N Engl J Med.

[7] Motzer RJ, Penkov K, Haanen J, Rini B, Albiges L, Campbell MT (2019). Avelumab plus Axitinib versus Sunitinib for Advanced Renal-Cell Carcinoma. N Engl J Med.

[8] Rini BI, Plimack ER, Stus V, Gafanov R, Hawkins R, Nosov D (2019). Pembrolizumab plus Axitinib versus Sunitinib for Advanced Renal-Cell Carcinoma. N Engl J Med.

[9] Mollica V, Di Nunno V, Gatto L, Santoni M, Scarpelli M, Cimadamore A et al. Resistance to systemic agents in renal cell Carcinoma Predict and overcome genomic strategies adopted by Tumor. Cancers. 2019;11(6). 10.3390/cancers11060830 PMC6627706 31207938

[10] Schaue D, McBride WH (2015). Opportunities and challenges of radiotherapy for treating cancer. Nat Reviews Clin Oncol.

[11] Deschavanne PJ, Fertil B (1996). A review of human cell radiosensitivity in vitro. Int J Radiation Oncology*Biology*Physics.

[12] Choi R, Yu JB (2019). Radiation Therapy for Renal Cell Carcinoma. Kidney Cancer.

[13] Wu Y, Song Y, Wang R, Wang T. Molecular mechanisms of tumor resistance to radiotherapy. Mol Cancer. 2023;22(1). 10.1186/s12943-023-01801-2 PMC10268375 37322433

[14] Siva S, Louie AV, Warner A, Muacevic A, Gandhidasan S, Ponsky L (2017). Pooled analysis of stereotactic ablative radiotherapy for primary renal cell carcinoma: a report from the International Radiosurgery Oncology Consortium for kidney (IROCK). Cancer.

[15] Wang H, Mu X, He H, Zhang X-D (2018). Cancer Radiosensitizers Trends Pharmacol Sci.

[16] Kuwahara Y, Mori M, Kitahara S, Fukumoto M, Ezaki T, Mori S (2014). Targeting of tumor endothelial cells combining 2 Gy/day of X-ray with Everolimus is the effective modality for overcoming clinically relevant radioresistant tumors. Cancer Med.

[17] Battelli C, Cho DC (2011). mTOR inhibitors in renal cell carcinoma. Therapy.

[18] Panwar V, Singh A, Bhatt M, Tonk RK, Azizov S, Raza AS et al. Multifaceted role of mTOR (mammalian target of rapamycin) signaling pathway in human health and disease. Signal Transduct Target Therapy. 2023;8(1). 10.1038/s41392-023-01608-z PMC10543444 37779156

[19] Deutsch E, Le Péchoux C, Faivre L, Rivera S, Tao Y, Pignon JP (2015). Phase I trial of everolimus in combination with thoracic radiotherapy in non-small-cell lung cancer. Ann Oncol.

[20] Vapiwala N, Narayan V, Subramanian P, Christodouleas JP, Bekelman JE, Mick R et al. Phase I trial of Everolimus in Combination with Salvage Radiation Therapy (RT) for post-prostatectomy biochemical recurrence (BCR) in prostate Cancer (PC) patients. Int J Radiation Oncology*Biology*Physics. 2016;96(2). 10.1016/j.ijrobp.2016.10.013 27986349

[21] Narayan V, Vapiwala N, Mick R, Subramanian P, Christodouleas JP, Bekelman JE (2017). Phase 1 trial of Everolimus and Radiation Therapy for Salvage Treatment of Biochemical Recurrence in prostate Cancer patients following prostatectomy. Int J Radiation Oncology*Biology*Physics.

[22] Detti B, Francolini G, Becherini C, Olmetto E, Giacomelli I, Scartoni D (2016). Complete response in metastatic renal cell carcinoma after radiotherapy and everolimus: a clinical case and review of the literature. J Chemother.

[23] Chinnaiyan P, Won M, Wen PY, Rojiani AM, Werner-Wasik M, Shih HA (2018). A randomized phase II study of everolimus in combination with chemoradiation in newly diagnosed glioblastoma: results of NRG Oncology RTOG 0913. Neurooncology.

[24] Ma DJ, Galanis E, Anderson SK, Schiff D, Kaufmann TJ, Peller PJ (2015). A phase II trial of everolimus, temozolomide, and radiotherapy in patients with newly diagnosed glioblastoma: NCCTG N057K. Neurooncology.

[25] Sarkaria JN, Galanis E, Wu W, Peller PJ, Giannini C, Brown PD (2011). North Central Cancer Treatment Group Phase I Trial N057K of Everolimus (RAD001) and Temozolomide in Combination with Radiation Therapy in patients with newly diagnosed Glioblastoma Multiforme. Int J Radiation Oncology*Biology*Physics.

[26] Carew JS, Espitia CM, Zhao W, Mita MM, Mita AC, Nawrocki ST (2015). Targeting Survivin inhibits renal cell carcinoma progression and enhances the activity of Temsirolimus. Mol Cancer Ther.

[27] Hu S, Fu S, Xu X, Chen L, Xu J, Li B (2015). The mechanism of radiosensitization by YM155, a Novel small molecule inhibitor of Survivin expression, is Associated with DNA damage repair. Cell Physiol Biochem.

[28] Albadari N, Li W. Survivin small molecules inhibitors: recent advances and challenges. Molecules. 2023;28(3). 10.3390/molecules28031376 PMC9919791 36771042

[29] Rödel F, Hoffmann L, Herrmann M, Noisternig T, Papadopoulos T (2005). Survivin as a Radioresistance Factor, and Prognostic and Therapeutic Target for Radiotherapy in rectal Cancer. Cancer Res.

[30] Chakravarti A, Zhai GG, Zhang M, Malhotra R, Latham DE, Delaney MA (2004). Survivin enhances radiation resistance in primary human glioblastoma cells via caspase-independent mechanisms. Oncogene.

[31] Asanuma K, Moriai R, Yajima T, Yagihashi A, Yamada M, Kobayashi D (2005). Survivin as a Radioresistance factor in pancreatic Cancer. Jpn J Cancer Res.

[32] Yang CT, Li JM, Weng HH, Li YC, Chen HC, Chen MF (2009). Adenovirus-mediated transfer of siRNA against survivin enhances the radiosensitivity of human non-small cell lung cancer cells. Cancer Gene Ther.

[33] Chen X, Duan N, Zhang C, Zhang W (2016). Survivin and Tumorigenesis: Molecular mechanisms and therapeutic strategies. J Cancer.

[34] Hagenbuchner J, Kuznetsov AV, Obexer P, Ausserlechner MJ (2012). BIRC5/Survivin enhances aerobic glycolysis and drug resistance by altered regulation of the mitochondrial fusion/fission machinery. Oncogene.

[35] Rivadeneira DB, Caino MC, Seo JH, Angelin A, Wallace DC, Languino LR et al. Survivin promotes oxidative phosphorylation, subcellular mitochondrial repositioning, and tumor cell invasion. Sci Signal. 2015;8(389). 10.1126/scisignal.aab1624 PMC4539531 26268608

[36] Li F, Aljahdali I, Ling X. Cancer therapeutics using survivin BIRC5 as a target: what can we do after over two decades of study? J Experimental Clin Cancer Res. 2019;38(1). 10.1186/s13046-019-1362-1 PMC6704566 31439015

[37] Aoyama Y, Katashima M, Sawamoto T (2012). Lack of differences in the pharmacokinetics of sepantronium bromide (YM155) between US and Japanese patients with advanced solid tumors or non-hodgkin lymphoma. Biopharm Drug Dispos.

[38] Mokhtari RB, Homayouni TS, Baluch N, Morgatskaya E, Kumar S, Das B (2017). Combination therapy in combating cancer. Oncotarget.

[39] Kydd J, Jadia R, Velpurisiva P, Gad A, Paliwal S, Rai P. Targeting strategies for the Combination treatment of Cancer using drug Delivery systems. Pharmaceutics. 2017;9(4). 10.3390/pharmaceutics9040046 PMC5750652 29036899

[40] Pal K, Madamsetty VS, Dutta SK, Mukhopadhyay D (2019). Co-delivery of everolimus and vinorelbine via a tumor-targeted liposomal formulation inhibits tumor growth and metastasis in RCC. Int J Nanomed.

[41] Pal K, Madamsetty VS, Dutta SK, Wang E, Angom RS, Mukhopadhyay D. Synchronous inhibition of mTOR and VEGF/NRP1 axis impedes tumor growth and metastasis in renal cancer. Npj Precision Oncol. 2019;3(1). 10.1038/s41698-019-0105-2 PMC6895165 31840081

[42] Karyampudi L, Lamichhane P, Scheid AD, Kalli KR, Shreeder B, Krempski JW (2014). Accumulation of memory precursor CD8 T cells in regressing tumors following combination therapy with vaccine and anti-PD-1 antibody. Cancer Res.

[43] Kremer EJ, Norian LA, Kresowik TP, Rosevear HM, James BR, Rosean TR et al. Eradication of metastatic renal cell carcinoma after Adenovirus-encoded TNF-Related apoptosis-inducing ligand (TRAIL)/CpG immunotherapy. PLoS ONE. 2012;7(2). 10.1371/journal.pone.0031085 PMC3270031 22312440

[44] Jaafar-Maalej C, Diab R, Andrieu V, Elaissari A, Fessi H (2009). Ethanol injection method for hydrophilic and lipophilic drug-loaded liposome preparation. J Liposome Res.

[45] Foucquier J, Guedj M. Analysis of drug combinations: current methodological landscape. Pharmacol Res Perspect. 2015;3(3). 10.1002/prp2.149 PMC4492765 26171228

[46] Duarte D, Vale N. Evaluation of synergism in drug combinations and reference models for future orientations in oncology. Curr Res Pharmacol Drug Discovery. 2022;3. 10.1016/j.crphar.2022.100110 PMC9127325 35620200

[47] Blanco E, Shen H, Ferrari M (2015). Principles of nanoparticle design for overcoming biological barriers to drug delivery. Nat Biotechnol.

[48] Honary S, Zahir F. Effect of Zeta Potential on the properties of Nano-Drug Delivery Systems - a review (part 1). Trop J Pharm Res. 2013;12(2).

[49] Li K-L, Wang Y-F, Qin J-R, Wang F, Yang Y-T, Zheng L-W et al. Rapamycin enhances the anti-angiogenesis and anti-proliferation ability of YM155 in oral squamous cell carcinoma. Tumor Biology. 2017;39(6). 10.1177/1010428317706213 28618939

[50] Danielpour D, Gao Z, Zmina PM, Shankar E, Shultes BC, Jobava R et al. Early Cellular responses of Prostate Carcinoma Cells to Sepantronium Bromide (YM155) involve suppression of mTORC1 by AMPK. Sci Rep. 2019;9(1). 10.1038/s41598-019-47573-y PMC6687778 31395901

[51] Huynh H, Ng WH, Soo KC. Everolimus acts in Synergy with Vinorelbine to suppress the growth of Hepatocellular Carcinoma. Int J Mol Sci. 2023;25(1). 10.3390/ijms25010017 PMC10779360 38203186

[52] Jansson-Löfmark R, Hjorth S, Gabrielsson J (2020). Does in Vitro Potency Predict clinically efficacious concentrations?. Clin Pharmacol Ther.

[53] Lane HA, Wood JM, McSheehy PMJ, Allegrini PR, Boulay A, Brueggen J (2009). mTOR inhibitor RAD001 (Everolimus) has Antiangiogenic/Vascular properties distinct from a VEGFR tyrosine kinase inhibitor. Clin Cancer Res.

[54] Tu SP, Jiang XH, Lin MC, Cui JT, Yang Y, Lum CT (2003). Suppression of survivin expression inhibits in vivo tumorigenicity and angiogenesis in gastric cancer. Cancer Res.

[55] Sia J, Szmyd R, Hau E, Gee HE. Molecular mechanisms of Radiation-Induced Cancer Cell death: a primer. Front Cell Dev Biology. 2020;8. 10.3389/fcell.2020.00041 PMC7031160 32117972

[56] Komorowska D, Radzik T, Kalenik S, Rodacka A. Natural Radiosensitizers in Radiotherapy: Cancer Treatment by combining Ionizing Radiation with Resveratrol. Int J Mol Sci. 2022;23(18). 10.3390/ijms231810627 PMC9501384 36142554

[57] Gong L, Zhang Y, Liu C, Zhang M, Han S (2021). Application of Radiosensitizers in Cancer Radiotherapy. Int J Nanomed.

[58] Weichselbaum RR, Liang H, Deng L, Fu Y-X (2017). Radiotherapy and immunotherapy: a beneficial liaison?. Nat Reviews Clin Oncol.

[59] Di Maggio FM, Minafra L, Forte GI, Cammarata FP, Lio D, Messa C et al. Portrait of inflammatory response to ionizing radiation treatment. J Inflamm. 2015;12(1). 10.1186/s12950-015-0058-3 PMC4336767 25705130

[60] Yoshimoto Y, Kono K, Suzuki Y (2015). Anti-tumor Immune responses Induced by Radiotherapy: a review. Fukushima J Med Sci.

[61] Walle T, Martinez Monge R, Cerwenka A, Ajona D, Melero I, Lecanda F. Radiation effects on antitumor immune responses: current perspectives and challenges. Therapeutic Adv Med Oncol. 2018;10. 10.1177/1758834017742575 PMC5784573 29383033

[62] Schaue D, Kachikwu EL, McBride WH (2012). Cytokines in Radiobiological responses: a review. Radiat Res.

[63] Demaria S, Formenti SC. Role of T lymphocytes in tumor response to radiotherapy. Front Oncol. 2012;2. 10.3389/fonc.2012.00095 PMC3426850 22937524

[64] Filatenkov A, Baker J, Mueller AMS, Kenkel J, Ahn GO, Dutt S (2015). Ablative Tumor Radiation can change the Tumor Immune Cell Microenvironment to induce durable complete remissions. Clin Cancer Res.

[65] Kang C, Jeong S-Y, Song SY, Choi EK (2020). The emerging role of myeloid-derived suppressor cells in radiotherapy. Radiation Oncol J.

[66] Kachikwu EL, Iwamoto KS, Liao Y-P, DeMarco JJ, Agazaryan N, Economou JS (2011). Radiation Enhances Regulatory T Cell Representation. Int J Radiation Oncology*Biology*Physics.

[67] Mondini M, Loyher P-L, Hamon P, Gerbé de Thoré M, Laviron M, Berthelot K (2019). CCR2-Dependent recruitment of Tregs and Monocytes following Radiotherapy is Associated with TNFα-Mediated resistance. Cancer Immunol Res.

[68] Huijts CM, Santegoets SJ, de Jong TD, Verheul HM, de Gruijl TD, van der Vliet HJ (2017). Immunological effects of everolimus in patients with metastatic renal cell cancer. Int J ImmunoPathol Pharmacol.

[69] Jutzy JMS, Khan S, Asuncion-Valenzuela MM, Milford T-AM, Payne KJ, Wall NR (2012). Tumor-released Survivin induces a Type-2 T cell response and decreases cytotoxic T cell function, in Vitro. Cancer Microenvironment.

[70] Chen H-y, Xu L, Li L-f, Liu X-x, Gao J-x. Bai Y-r. inhibiting the CD8 + T cell infiltration in the tumor microenvironment after radiotherapy is an important mechanism of radioresistance. Sci Rep. 2018;8(1). 10.1038/s41598-018-30417-6 PMC6085329 30093664

[71] Gupta A, Probst HC, Vuong V, Landshammer A, Muth S, Yagita H (2012). Radiotherapy promotes Tumor-Specific Effector CD8 + T cells via dendritic cell activation. J Immunol.

[72] Lee Y, Auh SL, Wang Y, Burnette B, Wang Y, Meng Y (2009). Therapeutic effects of ablative radiation on local tumor require CD8 + T cells: changing strategies for cancer treatment. Blood.

[73] Vakifahmetoglu H, Olsson M, Zhivotovsky B (2008). Death through a tragedy: mitotic catastrophe. Cell Death Differ.

[74] Mc Gee MM (2015). Targeting the mitotic catastrophe signaling pathway in Cancer. Mediat Inflamm.

[75] Vader G, Kauw JJW, Medema RH, Lens SMA (2006). Survivin mediates targeting of the chromosomal passenger complex to the centromere and midbody. EMBO Rep.

[76] Mita AC, Mita MM, Nawrocki ST, Giles FJ, Survivin (2008). Key Regulator of mitosis and apoptosis and Novel Target for Cancer therapeutics. Clin Cancer Res.

[77] Bai Z, Zhou Y, Ye X, Li Y, Peng Y, Guan Q et al. Survivin suppression heightens BZML-induced mitotic catastrophe to overcome multidrug resistance by removing therapy-induced senescent A549/Taxol cells. Biochimica et Biophysica Acta (BBA) -. Mol Cell Res. 2022;1869(2). 10.1016/j.bbamcr.2021.119174 34808206

[78] Martini E, Wittkopf N, Günther C, Leppkes M, Okada H, Watson Alastair J (2016). Loss of Survivin in Intestinal epithelial progenitor cells leads to mitotic catastrophe and breakdown of Gut Immune Homeostasis. Cell Rep.

[79] Lamers F, van der Ploeg I, Schild L, Ebus ME, Koster J, Hansen BR (2011). Knockdown of survivin (BIRC5) causes apoptosis in neuroblastoma via mitotic catastrophe. Endocrine-related Cancer.

[80] Niida H, Tsuge S, Katsuno Y, Konishi A, Takeda N, Nakanishi M (2005). Depletion of Chk1 leads to premature activation of Cdc2-cyclin B and mitotic catastrophe. J Biol Chem.

[81] Castedo M, Perfettini J-L, Roumier T, Yakushijin K, Horne D, Medema R (2004). The cell cycle checkpoint kinase Chk2 is a negative regulator of mitotic catastrophe. Oncogene.

[82] Huang T-T, Brill E, Nair JR, Zhang X, Wilson KM, Chen L (2020). Targeting the PI3K/mTOR pathway augments CHK1 inhibitor–Induced replication stress and Antitumor Activity in High-Grade Serous Ovarian Cancer. Cancer Res.

[83] Xu C, Gao Q, Wu Z, Lou W, Li X, Wang M et al. Combined HASPIN and mTOR inhibition is synergistic against KRAS-driven carcinomas. Translational Oncol. 2022;26. 10.1016/j.tranon.2022.101540 PMC9483799 36115073

