# Supplementary material for: Targeting mTOR and survivin concurrently potentiates radiation therapy in renal cell carcinoma by suppressing DNA damage repair and amplifying mitotic catastrophe
Source: J Exp Clin Cancer Res. 2024 Jun 6;43:159. doi: 10.1186/s13046-024-03079-8 (PMC11155143; doi:10.1186/s13046-024-03079-8)
Supplement: Supplementary file 1 — Supplementary Material 1 [file 13046_2024_3079_MOESM1_ESM.docx]

| **Liposome** | **DOPC (mg/mL)** | **Cholesterol (mg/mL)** | **DSPE-Peg(2000)-OMe (mg/mL)** | **TTP (mg/mL)** | **Everolimus (mg/mL)** | **YM155**  **(mg/mL)** | **DLE (%)** | **EE (%)** |
| --- | --- | --- | --- | --- | --- | --- | --- | --- |
| E-L | 3.93 | 0.483 | 0.27 | 0.22 | 0.393 ± 0.008 | --- | 7.42 ± 0.16 | 98.19 ± 2.13 |
| Y-L | 3.93 | 0.483 | 0.27 | 0.22 | --- | 0.297 ± 0.014 | 5.71 ± 0.26 | 37.14 ± 1.70 |
| EY-L | 3.93 | 0.483 | 0.27 | 0.22 | 0.387 ± 0.008 | 0.288 ± 0.019 | 6.94 ± 0.14 (E),  5.17 ± 0.34 (Y) | 96.73 ± 2.01 (E),  36.05 ± 2.35 (Y) |

**Supplementary Table S1: Composition of E-L, Y-L, and EY-L formulations.**
