# Supplementary material for: Targeting mTOR and survivin concurrently potentiates radiation therapy in renal cell carcinoma by suppressing DNA damage repair and amplifying mitotic catastrophe
Source: J Exp Clin Cancer Res. 2024 Jun 6;43:159. doi: 10.1186/s13046-024-03079-8 (PMC11155143; doi:10.1186/s13046-024-03079-8)
Supplement: Supplementary file 2 — Supplementary Material 2 [file 13046_2024_3079_MOESM2_ESM.docx]

| **Liposome** | **Hydrodynamic diameter (nm)** | **PDI** | **Zeta Potential (mV)** |
| --- | --- | --- | --- |
| E-L | 62.15 ± 0.40 | 0.178 ± 0.015 | 10.23 ± 2.4 |
| Y-L | 67.55 ± 0.24 | 0.195 ± 0.007 | 32.7 ± 4 |
| EY-L | 67.15 ± 0.31 | 0.205 ± 0.01 | 37.5 ± 3.3 |

**Supplementary Table S2: Characterization of E-L, Y-L, and EY-L formulations.**
