# Supplementary material for: Targeting mTOR and survivin concurrently potentiates radiation therapy in renal cell carcinoma by suppressing DNA damage repair and amplifying mitotic catastrophe
Source: J Exp Clin Cancer Res. 2024 Jun 6;43:159. doi: 10.1186/s13046-024-03079-8 (PMC11155143; doi:10.1186/s13046-024-03079-8)
Supplement: Supplementary file 3 — Supplementary Material 3 [file 13046_2024_3079_MOESM3_ESM.docx]

|  | **Bliss combination index of EY-L**  **over E-L and Y-L** |
| --- | --- |
| **786-O** | 0.809 |
| **Renca** | 0.503 |

**Supplementary Table S3: Bliss combination index of EY-L over E-L and Y-L in inducing radiosensitivity in 786-O and Renca cells.**
