# Supplementary material for: Targeting mTOR and survivin concurrently potentiates radiation therapy in renal cell carcinoma by suppressing DNA damage repair and amplifying mitotic catastrophe
Source: J Exp Clin Cancer Res. 2024 Jun 6;43:159. doi: 10.1186/s13046-024-03079-8 (PMC11155143; doi:10.1186/s13046-024-03079-8)
Supplement: Supplementary file 4 — Supplementary Material 4 [file 13046_2024_3079_MOESM4_ESM.pdf]

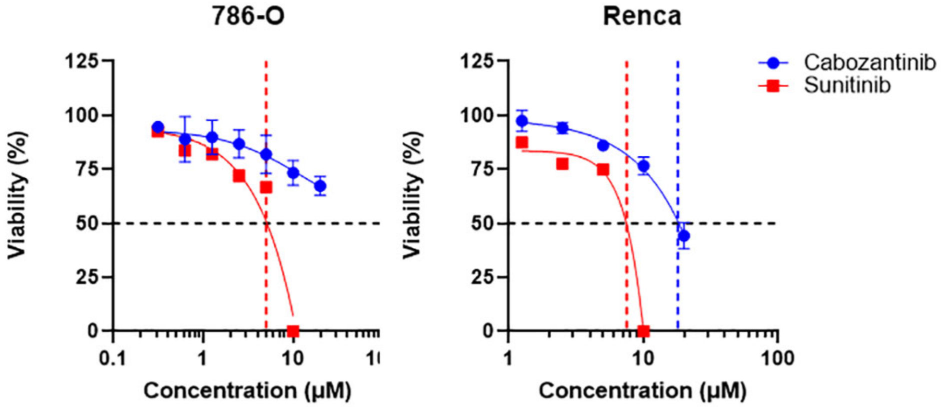

Supplementary Fig. S1. In vitro cytotoxicity of cabozantinib and sunitinib in 786-O and Renca cell line. MTS assay in 786-O and Renca cells treated with increasing concentrations of cabozantinib and sunitinib for 72 hours (n=4 wells per treatment condition).

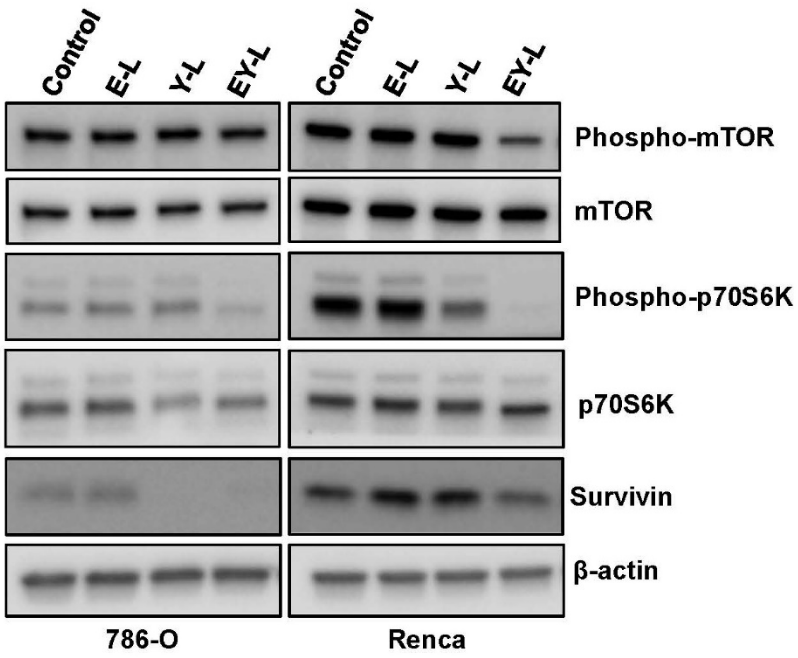

Supplementary Fig. S2. EY-L inhibits p70S6K phosphorylation and survivin expression. 786-O and Renca cells were treated for sub-IC50 concentrations of E-L, Y-L, and EY-L (0.01% liposomes for 786-O, 0.125% liposomes for Renca) for 48 hours. Cells were then harvested and lysed, and Western Blot analysis was employed to determine alterations in expressions and/or phosphorylations of mTOR, p70S6K, and survivin. β-actin was used as a loading control. EY-L showed strong inhibition of p70S6K phosphorylation and survivin expression and demonstrated a synergistic effect over E-L or Y-L in most cases.

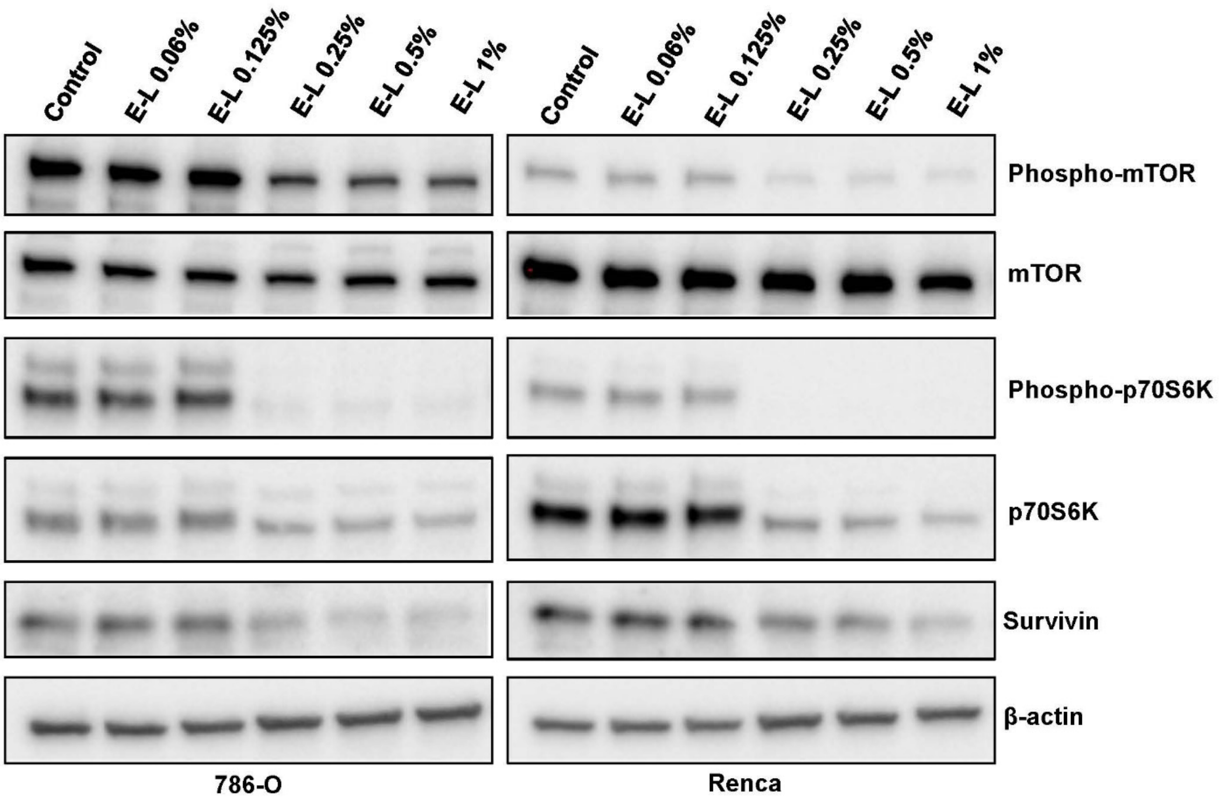

Supplementary Fig. S3. E-L inhibits mTOR signaling and survivin expression at higher concentrations. 786-O and Renca cells were treated with 0.06%, 0.125%, 0.25%, 0.5%, and 1% E-L for 48 hours. Cells were then harvested and lysed, and Western Blot analysis was employed to determine alterations in expressions or phosphorylations of mTOR, p70S6K, and survivin. β-actin was used as a loading control. E-L showed strong inhibition of phospho-mTOR, phospho-p70S6K, p70S6K, and survivin at concentrations starting from 0.25%.

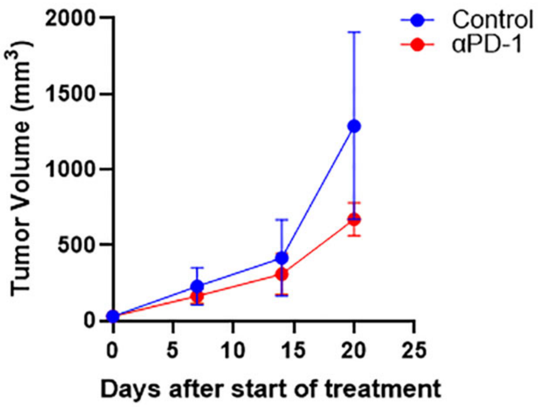

Supplementary Fig. S4. Growth curves for subcutaneous Renca tumors treated with a mouse anti-PD-1 antibody (αPD-1, 200 µg/mouse, every three to four days for a total of six treatments, n=5 mice per group).

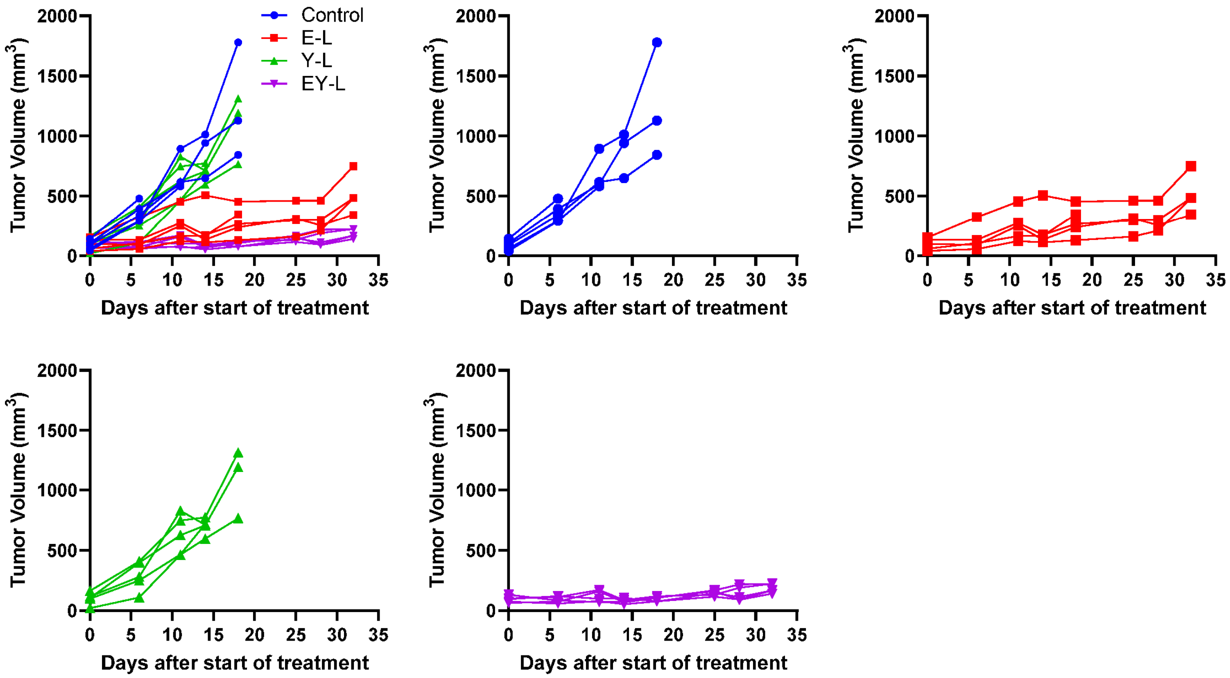

Supplementary Fig. S5. Individual tumor growth curves for Fig. 2A

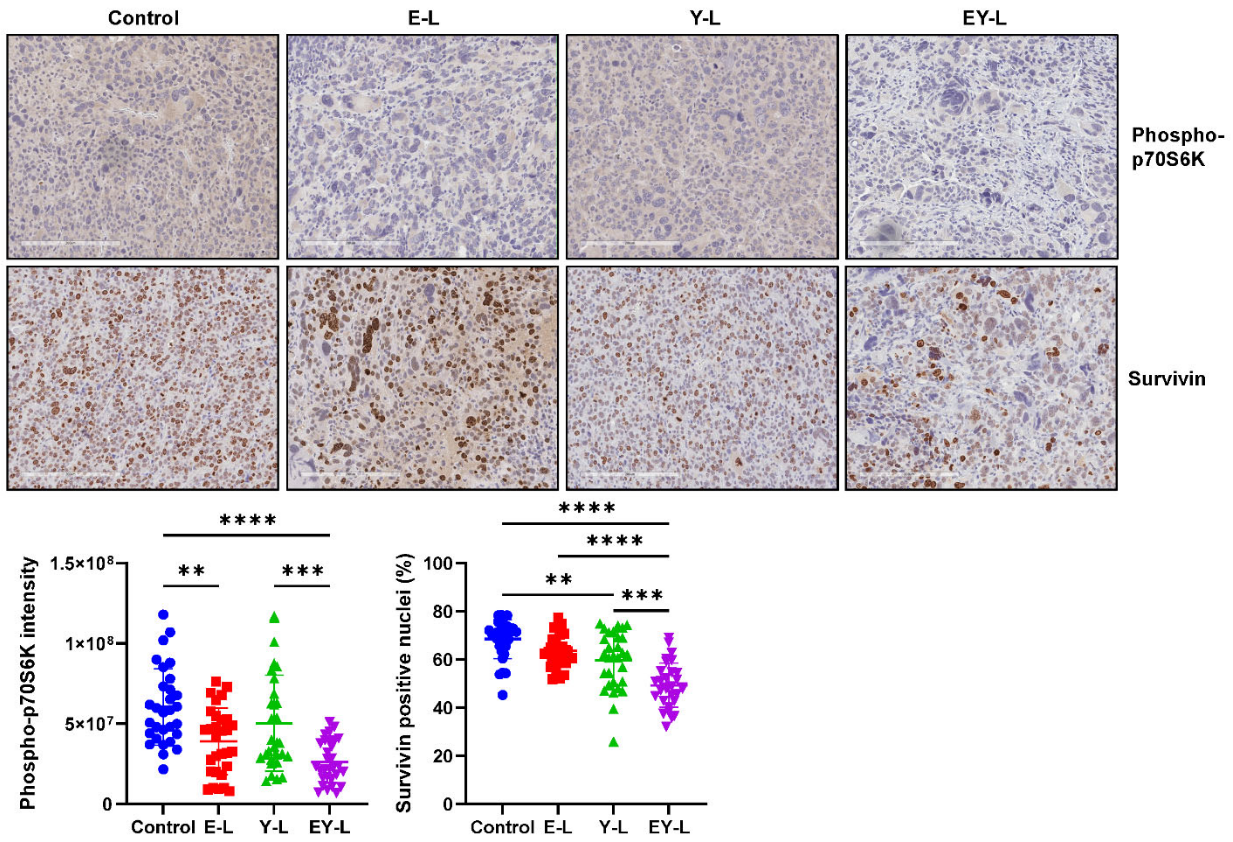

Supplementary Fig. S6. EY-L inhibits phospho-P70S6K and survivin in vivo. Representative images of phospho-p70S6K and survivin stained tumor sections and corresponding quantification (n=30, 10 visual fields 0.25 mm^2 each from 3 different tumor sections per group) from the experiment shown in figure 2A. Bar length = 200 µm. ** p<0.01, *** p<0.001, **** p<0.0001.

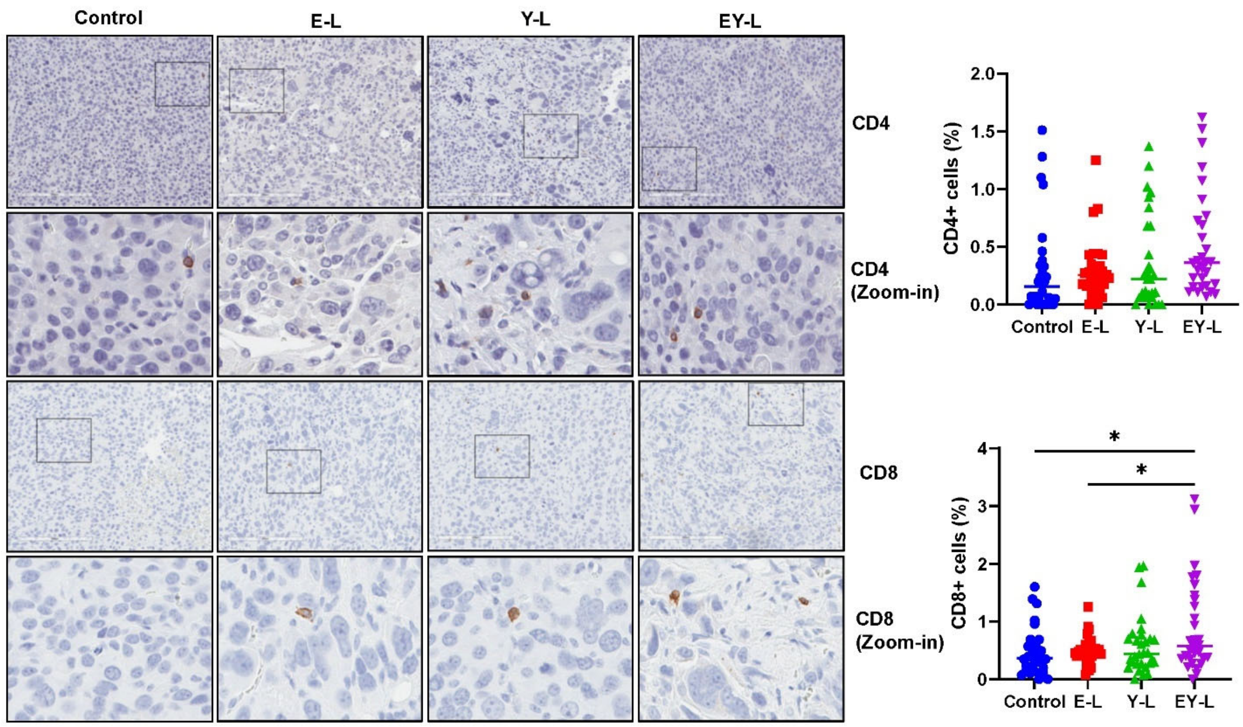

Supplementary Fig. S7. EY-L treatment leads to an increase in CD8+T cell infiltration in Renca tumors. Representative images of CD4 and CD8 stained Renca tumor sections and corresponding quantification (n=30, 10 visual fields 0.25 mm^2 each from 3 different tumor sections per group) from the experiment shown in figure 2A. Bar length = 200 µm. * $p<0.05$. Zoom-in images show the magnified image of the boxed regions from the corresponding original image.

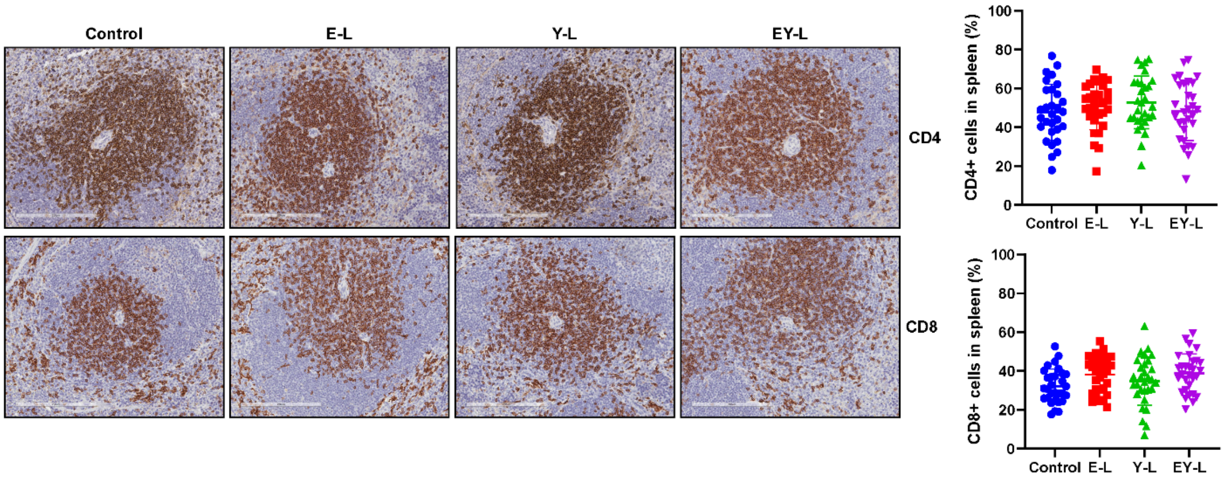

Supplementary Fig. S8. EY-L treatment does not significantly affect CD4+ and CD8+ T-cell abundance in the spleen. Representative images of CD4 and CD8 stained spleen T-cell zones and corresponding quantification (n=30, 10 visual fields 0.25 mm^2 each covering a T-cell zone from 3 different spleens per group) from the experiment shown in figure 2A. Bar length = 200 µm.

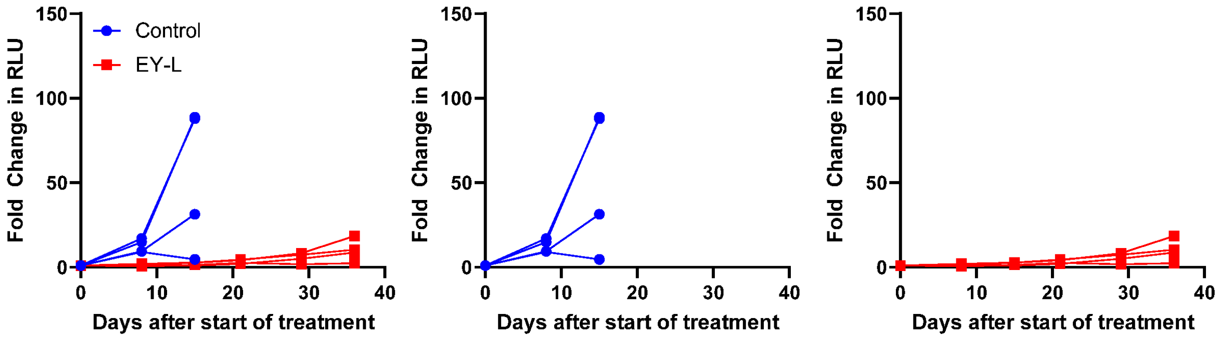

Supplementary Fig. S9. Individual tumor growth curves for Fig. 3B.

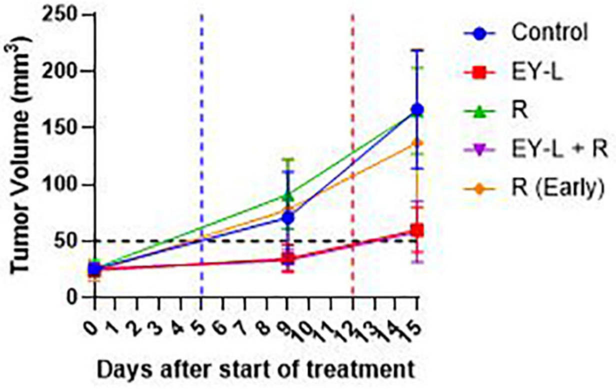

Supplementary Fig. S10. A zoomed-in portion of Fig. 4B demonstrates that the Day 5 tumor volume of the R(Early) group is equal to the Day 12 tumor volume of the EY-L+R group.

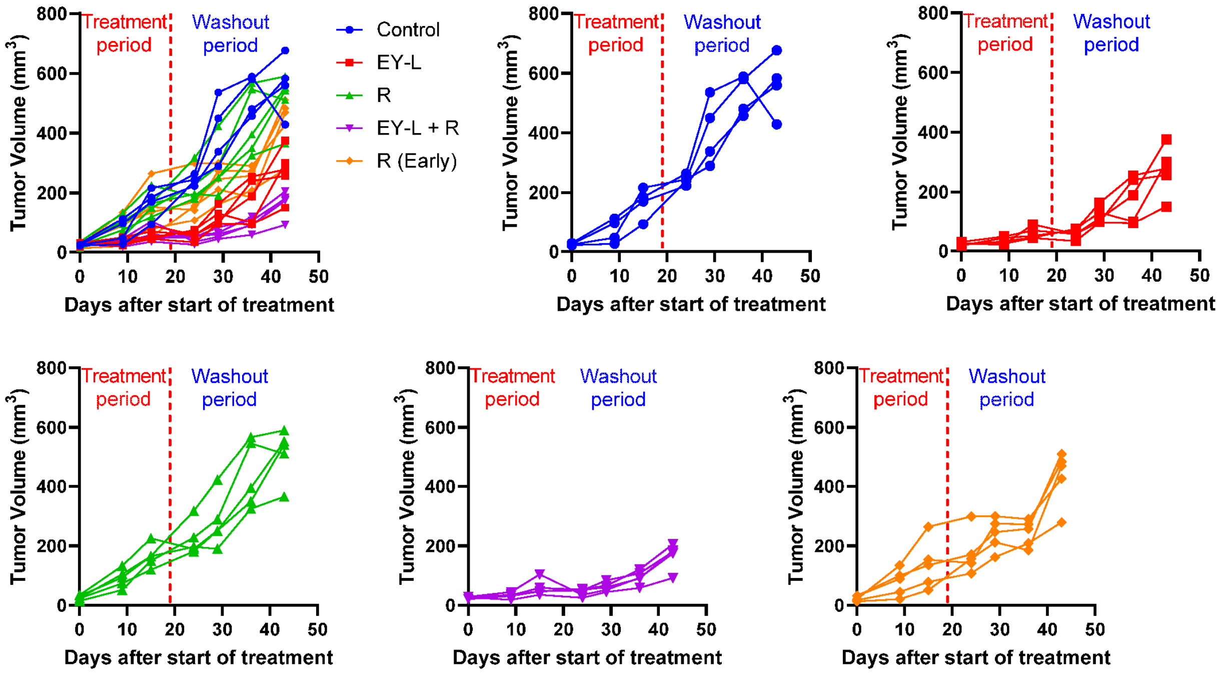

Supplementary Fig. S11. Individual tumor growth curves for Fig. 4B.

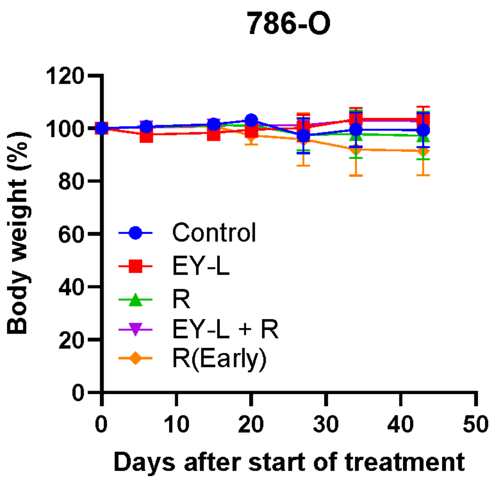

Supplementary Fig. S12. Body weight changes in the experiment shown in Fig. 4B.

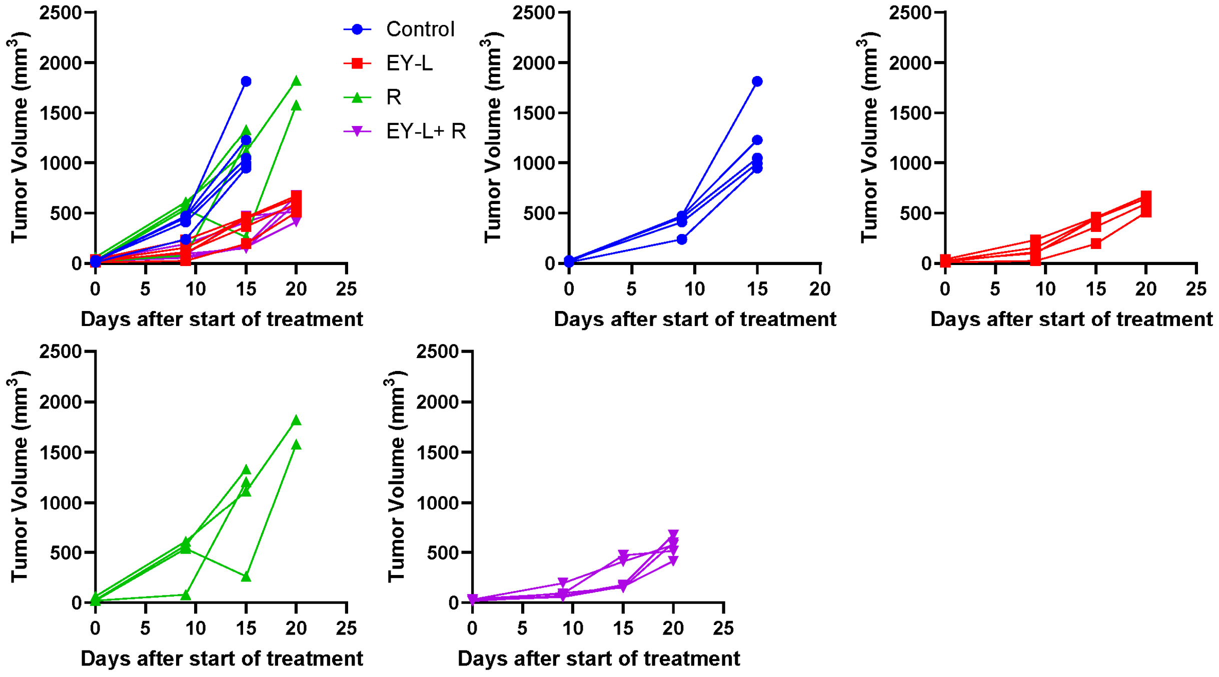

Supplementary Fig. S13. Individual tumor growth curves for Fig. 4C.

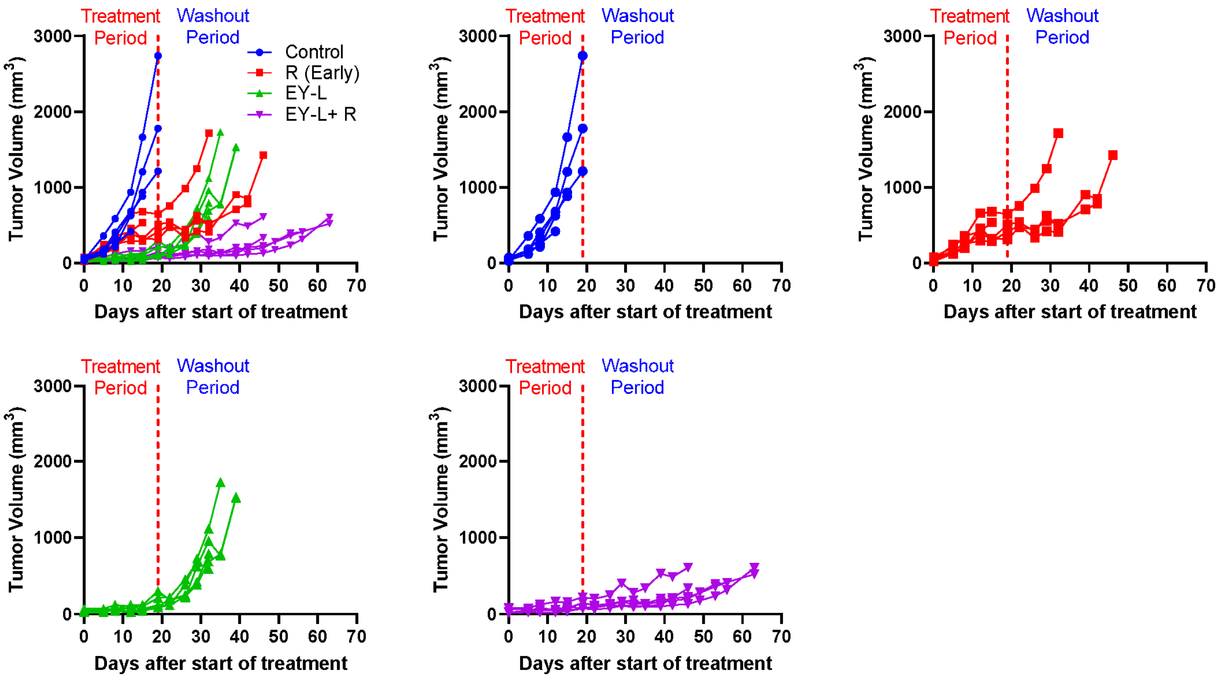

Supplementary Fig. S14. Individual tumor growth curves for Fig. 5B.

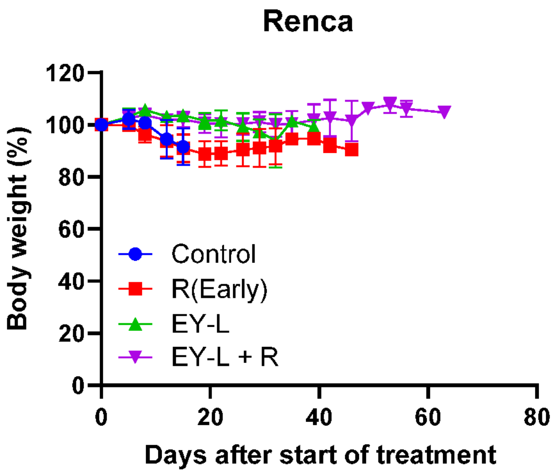

Supplementary Fig. S15. Body weight changes in the experiment shown in Fig. 5B.

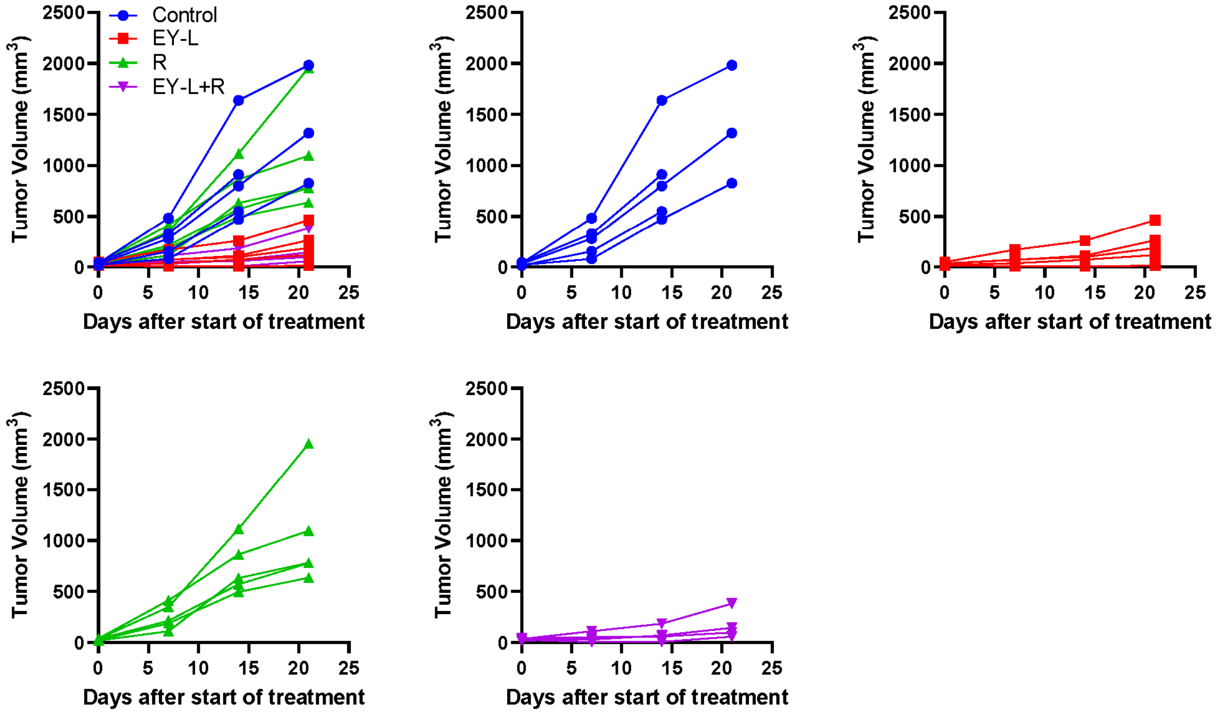

Supplementary Fig. S16. Individual tumor growth curves for Fig. 5C.

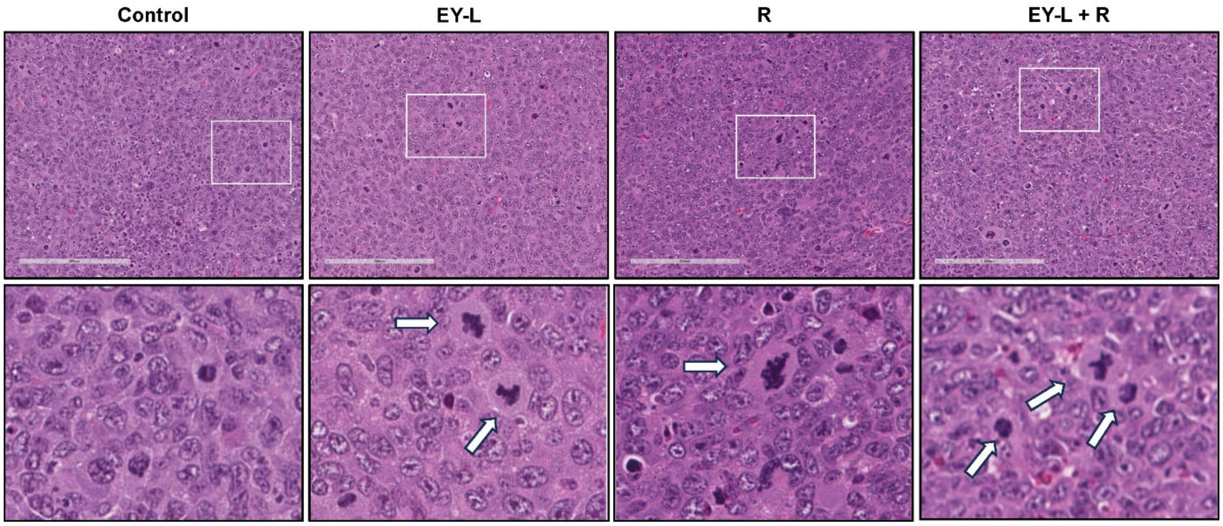

Supplementary Fig. S17. Mitotic catastrophe in 786-O xenografts treated with EY-L, radiation, or their combination. Tumor sections from the experiment shown in Fig. 4B were analyzed for the presence of mitotic catastrophe. While mitotic catastrophe could be seen in 786-O tumors treated with EY-L, radiation, or their combination, the effect is much less in 786-O xenografts than in Renca tumors, suggesting a plausible role of the immune system in mitotic catastrophe.
